# Feedback Activation of Basic Fibroblast Growth Factor Signaling via the Wnt/β-Catenin Pathway in Skin Fibroblasts

**DOI:** 10.3389/fphar.2017.00032

**Published:** 2017-02-03

**Authors:** Xu Wang, Yuting Zhu, Congcong Sun, Tao Wang, Yingjie Shen, Wanhui Cai, Jia Sun, Lisha Chi, Haijun Wang, Na Song, Chao Niu, Jiayi Shen, Weitao Cong, Zhongxin Zhu, Yuanhu Xuan, Xiaokun Li, Litai Jin

**Affiliations:** ^1^Key Laboratory of Biotechnology Pharmaceutical Engineering, School of Pharmaceutical Sciences, Wenzhou Medical UniversityWenzhou, China; ^2^Haining Hospital of Traditional Chinese MedicineHaining, China; ^3^School of Basic Medical Sciences, Xinxiang Medical UniversityXinxiang, China

**Keywords:** cell migration, bFGF, transcriptome, Wnt signaling pathway, β-catenin, GSK3β

## Abstract

Skin wound healing is a complex process requiring the coordinated behavior of many cell types, especially in the proliferation and migration of fibroblasts. Basic fibroblast growth factor (bFGF) is a member of the FGF family that promotes fibroblast migration, but the underlying molecular mechanism remains elusive. The present RNA sequencing study showed that the expression levels of several canonical Wnt pathway genes, including *Wnt2b*, *Wnt3*, *Wnt11*, *T-cell factor 7 (TCF7)*, and *Frizzled 8* (*FZD8*) were modified by bFGF stimulation in fibroblasts. Enzyme-linked immunosorbent assay (ELISA) analysis also showed that Wnt pathway was activated under bFGF treatment. Furthermore, treatment of fibroblasts with lithium chloride or IWR-1, an inducer and inhibitor of the Wnt signaling pathway, respectively, promoted and inhibited cell migration. Also, levels of cytosolic glycogen synthase kinase 3 beta phosphorylated at serine^9^ (pGSK3β Ser^9^) and nuclear β-catenin were increased upon exposure to bFGF. Molecular and biochemical assays indicated that phosphoinositide 3-kinase (PI3K) signaling activated the GSK3β/β-catenin/Wnt signaling pathway via activation of c-Jun N-terminal kinase (JNK), suggesting that PI3K and JNK act at the upstream of β-catenin. In contrast, knock-down of β*-catenin* delayed fibroblast cell migration even under bFGF stimulation. RNA sequencing analysis of β*-catenin* knock-down fibroblasts demonstrated that β-catenin positively regulated the transcription of *bFGF* and *FGF21.* Moreover, FGF21 treatment activated AKT and JNK, and accelerated fibroblast migration to a similar extent as bFGF does. In addition, ELISA analysis demonstrated that both of bFGF and FGF21 were auto secretion factor and be regulated by Wnt pathway stimulators. Taken together, our analyses define a feedback regulatory loop between bFGF (FGF21) and Wnt signaling acting through β-catenin in skin fibroblasts.

## Introduction

Skin serves as a barrier to protect internal tissues from the external environment, playing a crucial role in life sustenance (Enoch and Price, 2004). Skin wounding can be caused by tears, cuts, or contusions (Pazyar et al., 2014), and presents as a split in the epidermis, as well as deeper punctures extending to the dermis, subcutaneous fat, fascia, muscles, or the bone itself (Enoch and Price, 2004). The net result is morbidity from loss of function, negative psychosocial effects from disfigurement, or even mortality from loss of the skin’s barrier function (Amini-Nik et al., 2014). Therefore, it is essential that any wound sustained by the skin is fully healed to restore homeostasis.

Wound healing is a complex biological process (Thamm et al., 2013) that requires the actions of various cell types, including keratinocytes, fibroblasts, endothelial cells, macrophages, and platelets. These cells undergo proliferation, migration, infiltration, and inflammatory events, in addition to stimulation by and activation of growth factors and ECM signaling molecules to rebuild the skin (Martin, 1997; Wagner and Wehrmann, 2007). Cell migration and proliferation are important processes that both require and trigger new ECM synthesis, thereby contributing to wound healing (Epstein et al., 1999). Fibroblast proliferation and migration are especially critical to the formation of granulation tissue and wound healing (Kanazawa et al., 2010).

Wound healing processes are regulated by numerous growth factors, including FGFs (Xuan et al., 2014). As one of the FGFs, bFGF/FGF2 is involved in many biological activities (e.g., cell growth, cell and tissue differentiation, and cell migration) (Dvorak and Hampl, 2005). Our previous studies reported that bFGF promoted wound closure through the PI3K/Rac1/JNK pathway and NFκB-JNK pathway (Xuan et al., 2014, 2016).

The canonical Wnt signaling pathway, also known as the β-catenin pathway or the β-catenin/ TCF pathway (Shtutman et al., 1999), likewise regulates a wide array of biological processes (Moon et al., 2004; Clevers, 2006; Brack et al., 2007). The hallmark of the Wnt/β-catenin pathway is the stabilization of cytosolic β-catenin. Under unstimulated conditions, β-catenin is constantly phosphorylated by a destruction complex consisting of GSK3β and other proteins (Gordon and Nusse, 2006); phosphorylated β-catenin is ubiquitinated by this complex and targeted for degradation by the proteasome (Aberle et al., 1997; Gordon and Nusse, 2006). Activation of the Wnt cascade inhibits GSK3β activity, allowing β-catenin to accumulate and enter the nucleus, where it associates with TCF/LEF, leading to the transcription of Wnt signaling genes that participate in cell survival, proliferation, and differentiation (Gordon and Nusse, 2006).

Previous study reported that FGF and Wnt/β-catenin signaling involved in collective cell migration (Aman and Piotrowski, 2008), additionally, Bergmann et al. (2011) also define the activation of Wnt pathway in fibroblast facilitate the release of collagen. Moreover, stimulation of osteoblast differentiation and bone formation is partially mediated by bFGF modulation of the Wnt pathway (Fei et al., 2011). However, the molecular basis for the relationship between bFGF and Wnt signaling during fibroblast migration is remained to be established.

Here, we performed an RNA sequencing analysis to gain insights into the molecular mechanism of bFGF-mediated wound healing, and identified 830 differentially expressed genes in bFGF-treated versus untreated fibroblasts. GO and pathway analyses distinguished a number of significantly altered molecular pathways, especially in the canonical Wnt signaling pathway. Moreover, GSK3β activity was decreased in response to bFGF treatment, while knock-down of β*-catenin* resulted in delay of cell migration. Lastly, suppression of β*-catenin* in fibroblasts led to repression of *bFGF* and *FGF21*, which detected with the identical effect on the activation of AKT and JNK, and the acceleration of cell migration.

To date, several studies have drawn attention to the role of FGF21 in modulation glucose homeostasis by binding to FGF receptor 1c isoform in the present of the cofactor β-klotho. Here, we report for the first time the involvement of FGF21 in fibroblast migration.

Taken together, our findings provide a new mechanism whereby bFGF (FGF21) and Wnt signaling are tightly connected to maintain fibroblast cell migration through β-catenin.

## Materials and Methods

### Ethics Statement

Human foreskin samples were collected from volunteers at the First Affiliated Hospital, Wenzhou Medical University. All volunteers were informed of the purpose and procedures of this study and agreed to offer their tissue specimens with written consent. All protocols for human studies were approved by the Ethics Committee of the First Affiliated Hospital of Wenzhou Medical University.

### Cell Culture

The NIH/3T3 mouse embryo fibroblast cell lines were purchased from American Type Cell Collection (ATCC, Manassas, VA, USA) and cultured in DMEM, which contained 5.5 mM glucose, 10% FBS and 1% penicillin-streptomycin. Cells passaged 3–15 times were selected for the following experiments.

While for primary human foreskin fibroblasts, fat was removed from all tissue samples, which were cut into 3 mm strips and incubated with 0.05% dispase neutral protease (Sigma-Aldrich, St. Louis, MO, USA). The tissues in digestion buffer were incubated with Dulbecco’s Modified Eagle’s Medium (DMEM; Gibco BRL, Grand Island, NY, USA) supplemented with 10% FBS (Gibco BRL) and 1% penicillin-streptomycin (Gibco BRL) at 4°C overnight. The epidermis was removed from the dermis, and the dermis was finely minced and placed into FBS-coated 25 cm^2^ flasks. The flasks were placed horizontally for 1 h and then vertically for 3 h in an atmosphere of 5% CO_2_ at 37°C. The tissues were then cultured in DMEM supplemented with 5.5 mM glucose, 10% FBS, and 1% penicillin-streptomycin, with subsequent changes of the medium every 3 days. Cultured cells were passaged using 0.25% trypsin (Gibco BRL) after cell confluence reached ∼80%. The human fibroblasts were used for various studies described below after 3–6 passages.

### Total RNA Extraction, cDNA Synthesis, and Quantitative Real-Time Polymerase Chain Reaction (qRT-PCR)

Total RNA was extracted from primary human fibroblasts cells with or without 1 h bFGF treatment (100 ng/mL) (Xuan et al., 2016). Our results showed that bFGF represses the protein levels of β-catenin in fibroblasts after 30 min of bFGF treatment (Supplementary Figure S1C). Therefore, total RNA was extracted from the mouse NIH3T3 fibroblast cell line with or without 30 min bFGF treatment (100 ng/mL), and β*-catenin* knock-down NIH3T3 cells for the RNA-Seq experiments. Cell monolayers were rinsed once with ice-cold phosphate-buffered saline, lysed directly in 3.5 cm culture dishes by adding 1 mL of Trizol Reagent (Invitrogen, Carlsbad, CA, USA) to each dish, and scraped with a cell scraper. Chloroform (0.2 mL) was added to 1 mL of cell lysate, and total RNA was extracted. Total RNA (2 μg) was reverse-transcribed using a GoScript Reverse Transcription Kit (Promega, Madison, WI, USA) according to the manufacturer’s instructions. Next, qRT-PCR was performed to quantify the expression of selected genes, as previously described (Zittermann and Issekutz, 2006). The mRNA levels were normalized against that of *GAPDH*. Gene-specific primer sequences used for qRT-PCR are listed in Supplementary Table S1.

### RNA Sequencing

Total RNA extracted from untreated or bFGF-treated (100 ng/mL, 1 h) human foreskin fibroblasts and β*-catenin* knock-down NIH3T3 cells were used for the RNA sequencing experiments. RNA sequencing was performed using a kit provided by LC Biotech, Co., Ltd (http://www.lc-bio.com/; Hangzhou, China), and data analyses were performed by Novel Bioinformatics, Co., Ltd (http://www.novelbio.com/; Shanghai, China).

### Analysis of GO Categories, Signaling Pathways, and Co-expression Networks

Differentially expressed genes were determined from statistical outcomes by testing for association with biological process GO terms (Consortium, 2006). Fisher’s exact test was used to classify the GO categories, and the false discovery rate (FDR) was calculated to correct the *P*-value, where the smaller the FDR, the smaller the error in judging the *P*-value (Dupuy et al., 2007). Enrichment of GO members among differentially expressed probe sets was identified using the one-tailed Fisher’s exact test according to 2 × 2 contingency tables (Dunnick et al., 2012). This provides a measure of the significance of the function. As the enrichment increases, the corresponding function is more specific, which helps in distinguishing GOs with a more concrete function.

Pathway analysis was used to determine the significance of assorted gene sets according to the Kyoto Encyclopedia of Genes and Genomes (KEGG), Biocarta, and Reactome databases. Fisher’s exact test was followed by Benjamini-Hochberg (BH) multiple testing correction to select the most significant pathway(s), and the threshold of significance was defined by the *P*-value and the FDR (Draghici et al., 2007).

### Wound Healing Assay

Cell migration was determined using a standard wound healing scratch assay. Cells were seeded into six-well plates and cultured overnight. Confluent cells were cultured in DMEM supplemented with 0.5% FBS for 24 h, and then wounded by creating a 1 mm linear scratch with a sterile pipette tip. Images of the wounded cell monolayers were taken under a Model IX70 Microscope (Olympus, Tokyo, Japan) at 0, 12, and 24 h after wounding. Cell migration into the wounded area was recorded for 24 h using the same microscope equipped with a CoolSNAP HQ CCD Camera (Nippon Roper, Chiba, Japan) and MetaMorph Software (Universal Imaging, Co., Ltd, Buckinghamshire, UK). All experiments were performed in the presence of 5 mg/mL mitomycin-C to inhibit cell proliferation. Concentrations of LiCl (Sigma) and IM-12 (Selleck), inhibitor of GSK3β and promotor of Wnt signaling, and IWR-1 (Sigma) and XAV-939 (Selleck), inhibitors of Wnt signaling, were tested and shown in Supplementary Figure S1. Concentrations of FGF21 and BGJ 398, an inhibitor of FGFR, were tested and shown in Supplementary Figure S7.

To observe the relationship between Wnt signaling and bFGF activation in cell migration, cells were pretreated with 1.0 μM IWR-1, 1.0 μM bFGF, or 1.0 μM IWR-1 plus 1.0 μM bFGF for 1 h before wounding. 24 h after wounding, the distance from 20 selected cells and the wound leading edge just after wounding was measured using Image J software (National Institutes of Health).

### Western Blot Analysis

Cells were harvested in an ice-cold lysis solution (7 M urea, 2 M thiourea, 2% CHAPS, 40 mM Tris base, 40 mM dithiothreitol, and 1% protease inhibitor) to procure whole cell extracts. KeyGen Nuclear-Cytosol Protein Extraction and KeyGen Mitochondria-Cytosol Protein Extraction Kits (Nanjing KeyGen Biotech., Co., Ltd, Nanjing, China) were used for protein isolation from the nucleus and the cytosol, respectively (Zhang et al., 2010). Protein concentrations were measured using the bicinchoninic acid protein assay reagent (Pierce/Life Technologies, Grand Island, NY, USA). Equal amounts of protein were loaded into each lane of a sodium dodecyl sulfate (SDS)-polyacrylamide gel, electrophoretically separated, and transferred onto a polyvinylidene difluoride (PVDF) membrane (Bio-Rad, Hercules, CA, USA). Membranes were blocked with 5% bovine serum albumin in Tris-buffered saline/Tween 20 (TBST) for 1 h and then incubated with the following primary antibodies overnight at 4°C: anti-GAPDH (Abcam, Cambridge, MA, USA), anti-β-actin (Cell Signaling Technology, Beverly, MA, USA), anti-β-catenin (Abcam), anti-phospho-GSK3β (pGSK3β Ser^9^) (Cell Signaling Technology), anti-GSK3β (Cell Signaling Technology), anti-pAKT Ser^473^ (Cell Signaling Technology), anti-total-AKT (Cell Signaling Technology), and anti-phospho-stress activated protein kinase (pSAPK)/JNK Thr^183^/Tyr^185^ (Cell Signaling Technology). After washing three times with TBST, blots were incubated with appropriate secondary antibodies (Cell Signaling Technology) at room temperature for 90 min. Finally, after washing with TBST, the antigen-antibody complexes on the PVDF membranes were visualized using an enhanced chemiluminescence kit (GE Healthcare, Piscataway, NJ, USA). Protein levels were normalized against GAPDH using Image J software, as previously described (Fei et al., 2011).

### Small Interfering RNA (siRNA) Experiments

The siRNA targeting the mouse *PI3-kinase p110α* (Cat. no. sc-39128) was purchased from Santa Cruz Biotechnology, Inc. (Santa Cruz, CA, USA). NIH3T3 cells were seeded 12 h before transfection, with a cell density of 30–50% confluence at the time of transfection. Next, the siRNA (40 nM) was transfected into the cells using Lipofectamine 2000 (Invitrogen) and Opti-MEMI reduced serum medium (Gibco BRL). After 24 h of transfection, cells were processed by semi-quantitative RT-PCR for the detection of *PI3K p110α* mRNA levels and Western blotting for the detection of p-GSK3β Ser^9^ levels.

### Silencing of Endogenous β-Catenin in Fibroblast Cells

The siRNA sequence used for the knock-down of β*-catenin* was as follows: 5′-GAACGCAGCAGCAGTTTGT-3′, as described by Singh et al. (2009), (nucleotides 124–142 on NM-007614.3). For lentiviral infection of NIH3T3 cells, cells were seeded at a density of 2 × 10^5^ cells per well in 24-well plates. After overnight culture, various amounts of lentivirus (3, 10, or 12 μL) (OBIO Technology, Co., Ltd, Shanghai, China) were added to the wells in the presence of 4 mg/mL Polybrene (Sigma-Aldrich, St. Louis, MO, USA). The plates were then centrifuged at 2,500 rpm at room temperature for 1 h and returned to the culture incubator. 24 h after infection, the resulting NIH3T3 cell cultures were analyzed for green fluorescence protein expression (GFP; a reporter) by confocal microscopy. Cells treated with 12 μL lentivirus were chosen for the knock-down experiments described below.

### Enzyme-Linked Immunosorbent Assay

Enzyme-linked immunosorbent assay was performed to detect the levels of FGFs (bFGF and FGF21, Multi Sciences, China) and Wnt3a (Enzyme-linked Biotechnology, Co., Ltd, Shanghai, China) according to the manufacturer’s instructions. Fibroblasts were seeded in 6-well plates and cultured for 24 h. To determine the assay of FGFs secreted by fibroblast, cell culture supernatants were collected after 24, 36, and 48 h, respectively. To determine the effect of β-catenin on FGFs secretion, after 24 h incubation, the media were replaced with fresh media containing 1.0 μM LiCl, 1.0 μM IWR-1, 0.5 μM IM-12 or 0.5 μM XAV-939, and the cells were then cultured for 48 h. To investigate Wnt3a secretion under FGFs treatment, fibroblasts were cultured in the fresh media containing 100 ng/mL bFGF or FGF21. Then, cell culture supernatants were collected to determine FGFs (bFGF and FGF21) and Wnt3a protein levels.

### ChIP Assay

Chromatin immunoprecipitation assay was performed by using a ChIP assay kit (cat no. 17-295, Millipore, Billerica, MA, USA) according to the manufacturer’s instructions. Cells were cross-linked with 1% formalin. DNA is extracted from immunoprecipitates of β-catenin Ab (Abcam, ab32572). For PCR, 2 uL from 30 uL of DNA extraction was used. The primer sequences for qRT-PCR are listed in Supplementary Table S1.

### Statistical Analysis

Statistical analysis was performed with GraphPad Prism 5 Software (GraphPad, San Diego, CA, USA). All data were expressed as mean ± the standard error (SE). Comparisons between two groups were performed using Student’s *t*-test.

## Results

### Identification of a bFGF-Regulated Transcriptome in Fibroblasts

**FIGURE 1:**
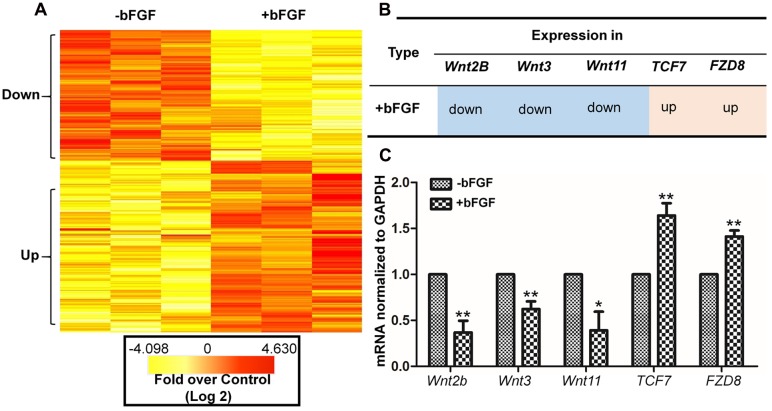
**Basic fibroblast growth factor-regulated Wnt signaling genes in skin fibroblasts. (A)** Heat map representation of genes whose expression levels were altered after 1 h of bFGF (100 ng/mL) treatment in human fibroblasts. Gene expression is shown by a pseudocolor scale, with yellow denoting low levels of gene expression, and red denoting high levels of gene expression (*P* < 0.05). **(B)** bFGF-regulated Wnt-related genes were classified into up-regulated and down-regulated clusters. **(C)** qRT-PCR was performed to monitor the mRNA levels of *FZD8*, *Wnt2b*, *Wnt11*, *Wnt3*, and *TCF7. GAPDH* was used as an internal control. Data represent mean values ± the SE (*n* = 5 replicates; ^∗^*P* < 0.05, ^∗∗^*P* < 0.01 versus the untreated control group; Student’s *t*-test).

To determine the effects of Wnt pathway gene expression after bFGF treatment, gene expression levels were analyzed using primary human fibroblast cells with or without bFGF treatment for 1 h (Xuan et al., 2016). RNA sequencing results revealed that 830 genes were differentially expressed in bFGF-treated fibroblasts. Among them, 473 genes were down-regulated, while 357 genes were up-regulated (*P* < 0.05 and fold change > 1.5) (**Figure 1A**) (Fei et al., 2011).

The significance of these altered gene expression levels was further assessed from statistical outcomes by testing for association with biological process GO terms. Pathway analysis was used to assess the significance of the differentially expressed gene sets according to KEGG, which demonstrated the following gene clusters that were significantly altered in bFGF-treated fibroblasts: seven up-regulated clusters (cytokine-cytokine receptor interactions, ECM-receptor interactions, transcriptional misregulation in cancer, human T-cell lymphotropic virus type 1 (HTLV-1) infection, *Salmonella* infection, hepatitis B, and hematopoietic cell lineage) and nine down-regulated clusters (Wnt signaling pathway, nuclear factor-kappa B signaling pathway, tumor necrosis factor signaling pathway, ECM-receptor interactions, Hippo signaling pathway, HTLV-1 infection, NOD-like receptor signaling pathway, Hedgehog signaling pathway, and insulin signaling pathway). As shown in **Figure 1B**, *Wnt2B*, *Wnt11*, and *Wnt3* were down-regulated, whereas *Frizzled 8* (*FZD8*) and *T-cell factor 7* (*TCF7*) were up-regulated in bFGF-treated fibroblasts. Furthermore, the changes in Wnt pathway genes (*FZD8*, *Wnt2B*, *Wnt11*, *Wnt3*, and *TCF7*) were verified by qRT-PCR, and the data were consistent with the RNA sequencing results (**Figure 1C**). These findings indicate that bFGF regulates Wnt signaling genes in fibroblasts.

### The Wnt/β-Catenin Pathway Positively Regulates Fibroblast Migration

**FIGURE 2:**
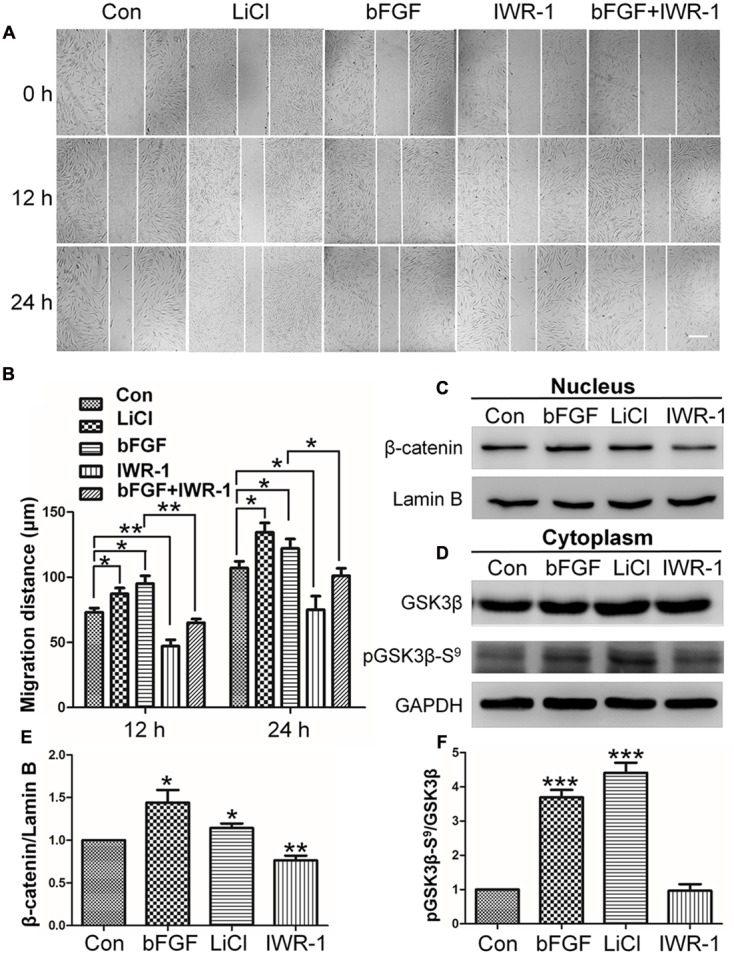
**Effects of LiCl, bFGF, and IWR-1 treatment on cell migration, nuclear β-catenin, and GSK3β phosphorylation levels in skin fibroblasts. (A)** A wound healing assay was performed after treatment with LiCl (1.0 μM), bFGF (100 ng/mL), IWR-1 (1.0 μM), or bFGF (100 ng/mL) plus IWR-1 (1.0 μM). The wounded cell monolayers were photographed at 0, 12, and 24 h after treatment. White vertical lines indicate the borders of the wound area. Bar = 500 μm. **(B)** Cell migration distances shown in **(A)** were measured and plotted (^∗^*P* < 0.05, ^∗∗^*P* < 0.01; Student’s *t*-test). **(C)** Nuclear β-catenin levels were altered upon treatment with bFGF, LiCl, or IWR-1. Preparation of nuclear lysates and Western blots were performed at 30 min (bFGF) or 1 h after each treatment. Nuclear lysates (20 μg of total protein) were loaded into each lane of the gel, electrophoresed, and transferred to a PVDF membrane. **(D)** Phosphorylation levels of GSK3β at Ser^9^ were altered by treatment with bFGF, LiCl, and IWR-1. The cells were stimulated with bFGF (100 ng/mL), LiCl (1 μM), or IWR-1 (1 μM) for 30 min or 1 h, and cell lysates (20 μg) were loaded into each lane of a SDS-polyacrylamide gel, electrophoresed, and transferred to a PVDF membrane. Densitometry data for β-catenin **(C)** or pGSK3β Ser^9^ **(D)** from the blot shown in **(E,F)** were normalized to those of Lamin B or GSK3β. The results are presented as fold changes relative to control fibroblasts grown in 5.5 mM glucose-containing DMEM. Data represent mean values ± the SE (*n* = 5 independent experiments; ^∗^*P* < 0.05, ^∗∗^*P* < 0.01, ^∗∗∗^*P* < 0.001; Student’s *t*-test). Con, control.

Since the expression levels of key Wnt signaling genes were significantly changed after bFGF treatment in skin fibroblasts, the role of Wnt signaling during human fibroblast migration was further analyzed. LiCl is an inhibitor of GSK3β (Klein and Melton, 1996) that activates the Wnt pathway, and accelerated cell migration herein throughout the course of the 24 h wound healing assay (**Figures 2A,B**; Supplementary Figure S1). By contrast, the Wnt pathway inhibitor, IWR-1 (Clevers and Nusse, 2012), delayed cell migration. Interestingly, bFGF accelerated migration of the fibroblasts, and slightly overturned the reduced cell migration caused by IWR-1 (**Figures 2A,B**). In addition, this critical effect of Wnt pathway on wound healing was further confirmed by IM-12 and XAV-939, another common used agonist and antagonist of Wnt signaling, respectively (Supplementary Figure S1) (Jeong et al., 2014; Cai et al., 2016).

### Effect of bFGF on Nuclear β-Catenin Accumulation and GSK3β Phosphorylation

The results from the cell migration assay indicated that Wnt signaling is important for skin wound healing. β-catenin is known as an essential transcription factor in the Wnt pathway that translocates from the cytosol to the nucleus to activate downstream target genes (Salic et al., 2000). Western blot analysis showed that bFGF or LiCl treatment increased nuclear β-catenin level, whereas IWR-1 reduced nuclear β-catenin level (**Figures 2C,E**).

To further analyze the potential mechanism by which bFGF regulates β-catenin degradation by proteasome, GSK3β phosphorylation levels were analyzed, because GSK3β phosphorylates β-catenin which further results in its degradation. On the other hand, GSK3β phosphorylation is reportedly involved in cell migration in various cellular systems (Etienne-Manneville and Hall, 2003; Bianchi et al., 2005; Sun et al., 2009; Harris and Nelson, 2010). Phosphorylation at Ser-9 inactivates GSK3β, which results in β-catenin stabilization and nuclear accumulation and leads to enhanced Wnt signaling (Fei et al., 2011). Therefore, we tested the effect of bFGF on GSK3β phosphorylation level. **Figures 2D,F** showed that bFGF and LiCl treatment increased the phosphorylation of GSK3β at Ser^9^ in fibroblasts, but no significant differences were observed between IWR-1-treated and control groups (Boku et al., 2013). These results suggested that bFGF inhibits GSK3β activity through increased phosphorylation of Ser^9^.

### bFGF/PI3K/JNK Signaling Activates the Wnt/β-Catenin Signaling Pathway

**FIGURE 3:**
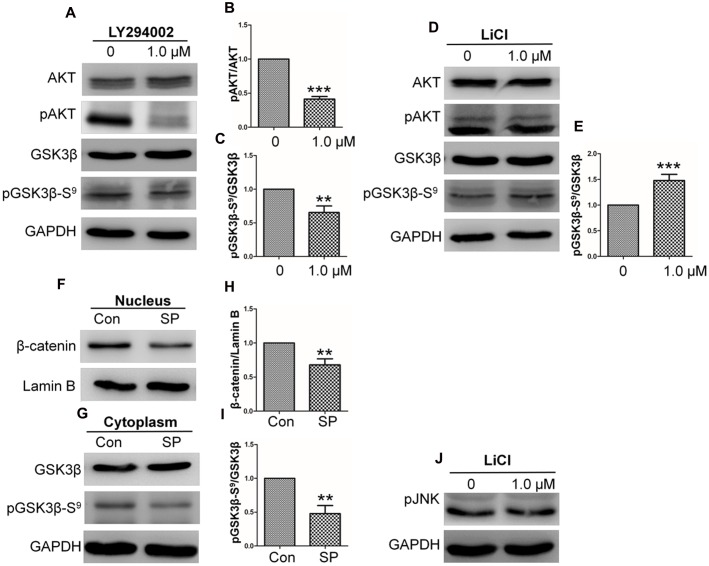
**Basic fibroblast growth factor activates the GSK3β/β-catenin/WNT signaling pathway via the PI3K/JNK pathway**. Human skin fibroblasts were treated for 1 h with the specific PI3K inhibitor, Ly294002 (1.0 μM), the GSK3β inhibitor, LiCl (1.0 μM), or solvent control. **(A)** Total cell lysates were then prepared and analyzed via Western blotting. Akt Ser^473^ and GSK3β Ser^9^ phosphorylation levels were blocked by Ly294002. **(D)** GSK3β Ser^9^ phosphorylation was increased by treatment with LiCl. **(F)** Nuclear β-catenin levels were increased by treatment with the JNK inhibitor, SP600125. **(G)** Phosphorylation levels of GSK3β at Ser^9^ were reduced by treatment with SP600125 for 1 h. **(J)** JNK phosphorylation was unchanged by LiCl treatment. Densitometry was performed on 3–4 Western blots (each representative of an independent experiment) per condition. Densitometry data for pAKT **(B)** or pGSK3β Ser^9^ **(C)** from the blots shown in **(A)** were normalized to those of AKT or GSK3β. Densitometry data for pGSK3β Ser^9^ **(E)** from the blot shown in **(D)** were normalized to those of GSK3β. Densitometry data for nuclear β-catenin **(H)** from the blot shown in **(F)** were normalized to those of Lamin B. Densitometry data for pGSK3β Ser^9^ **(I)** from the blot shown in **(F)** were normalized to those of GSK3β. The results are presented as fold changes relative to control fibroblasts grown in 5.5 mM glucose-containing DMEM. Data represent mean values ± the SE (*n* = 5 independent experiments; ^∗^*P* < 0.05, ^∗∗^*P* < 0.01, ^∗∗∗^*P* < 0.001; Student’s *t*-test). Con, control.

The PI3K/Rac1/JNK pathway functions at the downstream of bFGF during fibroblast migration (Kanazawa et al., 2010). Moreover, PI3K induces phosphorylation of AKT at Ser^473^, thereby facilitating the phosphorylation of GSK3β at Ser^9^, which in turn inactivates GSK3β kinase activity (Cohen and Frame, 2001; Engelman et al., 2006). To verify the relationship between PI3K-AKT and GSK3β in fibroblast, biochemical and pharmacological tests were further performed. Application of the PI3K inhibitor, LY294002 (cell signaling technology), substantially suppressed AKT and GSK3β phosphorylation levels in fibroblasts (**Figures 3A–C**). Contrarily, LiCl treatment did not change pAKT levels (**Figure 3D**), while pGSK3β Ser^9^ levels were increased (**Figure 3E**). In view of the fact that bFGF induced the neuroblast migration is mediated by modulating FGFR1 signaling via the PI3K *P110 alpha* isoform, specifically through the phosphorylation of PI3K downstream effectors, AKT and GSK3β (Hu et al., 2013), PI3K *P110 alpha* siRNA was transformed into NIH3T3 cells and PI3K levels were monitored. The data showed that both siRNA-mediated suppression of *PI3K* (*P110 alpha*) (Fukui et al., 2010) and neutralization of bFGF in the culture medium by a bFGF antibody reduced GSK3β Ser^9^ phosphorylation levels (Supplementary Figure S2).

JNK is a downstream regulator of PI3K that is reportedly involved in bFGF-regulated fibroblast migration (Kanazawa et al., 2010). In next, role of JNK in regulation of GSK3β phosphorylation was tested. The results showed that significant decrease of β-catenin and pGSK3β Ser^9^ were found in human fibroblasts treated with SP600125, a JNK inhibitor (**Figures 3F–I**). However, JNK phosphorylation levels did not differ with LiCl treatment (**Figure 3J**). Taken together, these results confirmed that bFGF/PI3K/JNK signaling acts at the upstream of GSK3β.

### β-Catenin Is Critical for bFGF-Mediated Cell Migration

**FIGURE 4:**
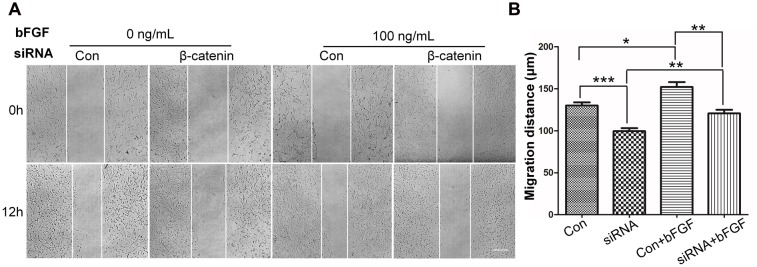
**Effect of siRNA-mediated inhibition of β-catenin in NIH3T3 cell migration. (A)** A wound healing assay was performed in cells transfected with scrambled control siRNA or β-catenin siRNA with or without bFGF (100 ng/mL). The wounded cell monolayers were photographed at 0 and 12 h after the treatment. White vertical lines indicate the borders of the wound area. Bar = 500 μm. **(B)** Cell migration distances shown in **(A)** were measured and plotted (^∗^*P* < 0.05, ^∗∗^*P* < 0.01, ^∗∗∗^*P* < 0.001; Student’s *t*-test). Con, control.

To determine the role of β-catenin in fibroblast migration, β*-catenin*-specific siRNA was used to repress β*-catenin* expression (Singh et al., 2009). Because human foreskin fibroblasts are active for a maximum of six generations or passages, they are not suitable for lentivirus-mediated transfection experiments. Thus, mouse NIH3T3 cells were employed for the siRNA experiments. The results showed that endogenous β-catenin levels in NIH3T3 cells were reduced by ∼50–95% following transfection (Supplementary Figure S3). Furthermore, β*-catenin* knock-down NIH3T3 cells exhibited a reduced rate of cell migration compared with control cells (**Figure 4A**), and LiCl or IWR-1 treatment exerted no effect on the β*-catenin* knock-down cell line (Supplementary Figure S4). Also, bFGF administration only slightly increased the migration rate of β*-catenin* knock-down cells (**Figure 4B**), indicating that β-catenin is necessary to promote bFGF-mediated fibroblast migration.

### β-Catenin Activates FGFs

**FIGURE 5:**
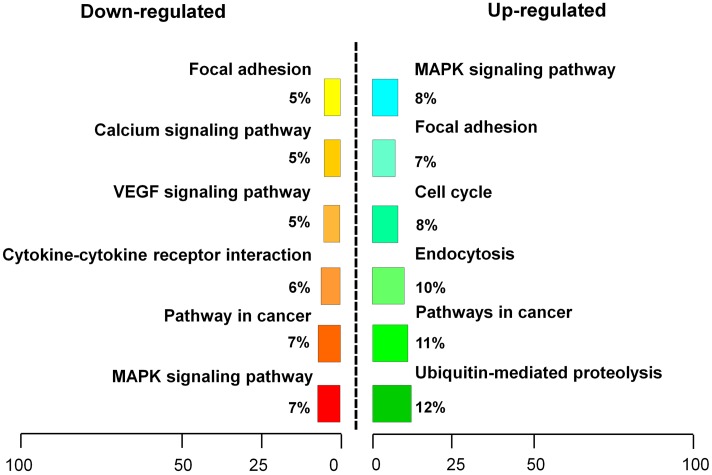
**Pathway analysis of β*-catenin*-regulating genes**. β*-catenin* suppression-regulating genes were classified into an up-regulated cluster and a down-regulated cluster.

**FIGURE 6:**
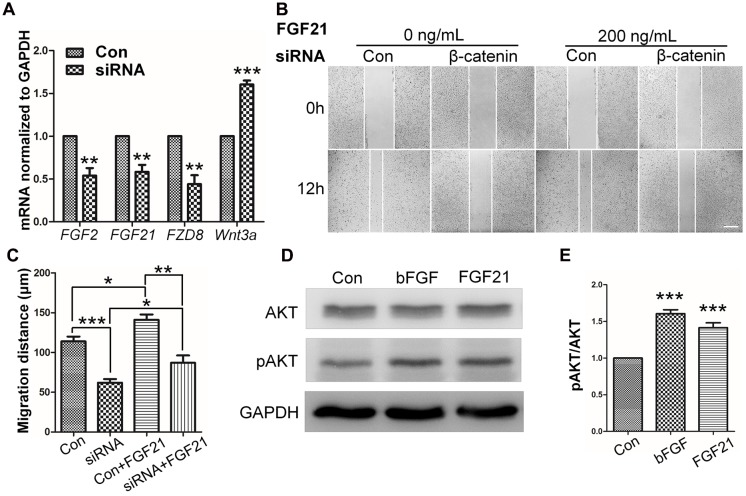
**Role of FGF and β-catenin in fibroblast migration and downstream regulations. (A)** qRT-PCR was performed to monitor the mRNA levels of *FGF2*, *FGF21*, *FZD8*, and *Wnt3a*. *GAPDH* was used as an internal control. Data represent mean values ± the SE of five replicates (*^∗∗^P* < 0.01, *^∗∗∗^P* < 0.0001 versus the untreated control group; Student’s *t*-test). **(B)** A wound healing assay was performed with or without FGF21 treatment (200 ng/mL), and the wounded cell monolayers were photographed 0 and 12 h later. White vertical lines indicate the borders of the wound area. Bar = 500 μm. **(C)** Cell migration distances were measured based on the data shown in **(B)**. Data represent mean values ± the SE of five replicates (*^∗^P* < 0.05, *^∗∗^P* < 0.01, *^∗∗∗^P* < 0.0001; Student’s *t*-test). **(D)** Western blotting was performed to analyze the protein levels of pAKT after 30 min treatment of fibroblasts with bFGF (100 ng/mL) or FGF21 (200 ng/mL). Densitometry data for pAKT **(E)** from the blot shown in **(D**) were normalized to those of AKT. All experiments were performed 24 h after application of 5 μg/mL mitomycin-C (a cell proliferation inhibitor). Data represent mean values ± the SE (*n* = 5 independent experiments; *^∗∗∗^P* < 0.0001 versus untreated control; Student’s *t*-test). Con, control.

RNA sequencing analysis was performed to examine transcriptome changes in β*-catenin* knock-down NIH3T3 cells. We found that 411 genes were differentially expressed in the β*-catenin* knock-down group. Among them, 201 genes were up-regulated, and 210 genes were down-regulated (*P* < 0.05 and fold change > 2.0) (Supplementary Table S2). These findings were further validated from statistical outcomes in tests for association with biological process GO terms (Supplementary Figure S5). Pathway analysis was used to determine the significance of the differentially expressed gene sets according to KEGG, which demonstrated that six up-regulated and six down-regulated clusters were significantly enriched by silencing of β*-catenin* (**Figure 5**). Interestingly, *FGF21* as well as *bFGF* expression levels were sensitive to β-catenin. According to the expression patterns summarized in **Figure 6A**, *bFGF* and *FGF21* were reduced significantly as well as Wnt pathway-related gene *FZD8*, and *Wnt3a* was increased by β*-catenin*-specific siRNA.

FGF21, a secreted protein (Badman et al., 2007), which is the most studied family member, and has been reported to be preferentially expressed in the liver early in development (Nishimura et al., 2000). However, recent studies have reported that FGF21 production is inducible by starvation or drug administration, and revealed its diverse functions in glucose homeostasis and protection of the liver and heart from injury (Lin et al., 2013, 2014; Liang et al., 2014).

To further understand the influence of *FGF21* in cell migration, wound healing scratch assay and Western blot were performed on NIH3T3 cells with FGF21 treatment. The results showed that FGF21 promoted cell migration of fibroblasts and β*-catenin*-suppressed NIH3T3 cells (**Figures 6B,C**; Supplementary Figures S6A–C). In addition, Western blot analysis showed that exogenous FGF21 treatment increased AKT phosphorylation levels (**Figures 6D,E**), and neutralization of FGF21 in the culture medium by a FGF21 antibody reduced GSK3β Ser^9^ phosphorylation levels to the same extent as bFGF (Supplementary Figures S6D,E). Taken together, these results suggest that FGF21 acted similarly to bFGF, therefore, it is necessary to further examine its roles in fibroblast cell migration.

### A Feedback Activation of bFGF via the Wnt/β-Catenin Pathway

**FIGURE 7:**
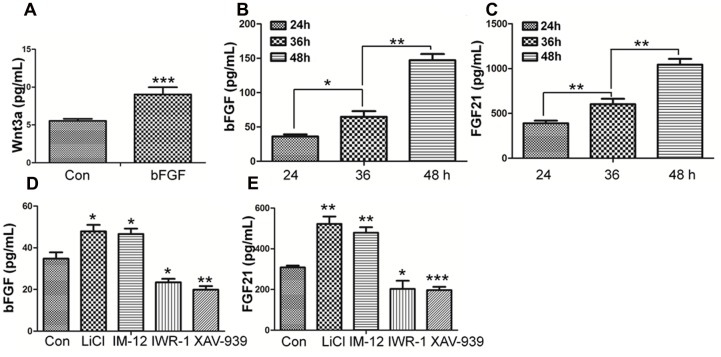
**Effect of bFGF on the secretion of Wnt3a and the Effects of normal condition and Wnt pathway agonists and antagonists on the secretion of bFGF and FGF21 in fibroblasts by ELISA. (A)** The cultured cells were treated with 100 ng/mL bFGF for 24 h, and the cell supernatants were collected to detect Wnt3a-secreted level. The results are presented the secretion of Wnt3a in fibroblasts is increased upon bFGF stimulation for 24 h. **(B,C)** The cells were cultured normally, and the cultured supernatants were collected after 24, 36, 48 h, respectively. The results are presented the release of bFGF and FGF21 in fibroblasts grown in 5.5 mM glucose-containing DMEM for 24 h. **(D,E)** The cultured cells were treated with 1.0 μM LiCl, 0.5 μM IM-12, 1.0 IWR-1, 0.5 μM XAV-939 or vehicle for 24 h, and the cell supernatants were collected to test the bFGF and FGF21 release. Elisa analysis showed that the secretion of bFGF and FGF21 in fibroblasts is up regulated upon LiCl and IM-12 stimulation for 24 h, while down regulated upon IWR-1 and XAV-939. Data represent mean values ± the SE (*n* = 5 independent experiments; ^∗^*P* < 0.05, ^∗∗^*P* < 0.01, ^∗∗∗^*P* < 0.001; Student’s *t*-test). Con, control.

Wnt3a, known as a main sign of activation of Canonical Wnt pathway, was assayed by ELISA (Shimizu et al., 1997). As shown in **Figure 7A**, Wnt3a in fibroblasts is increased upon bFGF stimulation, which further confirmed that Wnt signaling output was increased by bFGF treatment. On the other hand, the secretion of bFGF and FGF21 were analyzed under normal condition, Wnt pathway agonist and antagonist treatment, respectively (**Figures 7B–E**). The results showed that the LiCl and IM-12 treatment increased secretion of bFGF and FGF21, but decreased by IWR-1 and XAV-939 treatment. Besides, cell migration was reduced by addition of neutralizing antibody of bFGF and FGF21 in the culture medium (Supplementary Figure S7A). Moreover, BGJ398, an inhibitor of FGFR (Costa et al., 2014), slowed migration dramatically, which suggested that a feedback regulatory loop between bFGF (FGF21) and Wnt signaling acting through β-catenin in skin fibroblasts.

### β-Catenin Directly Activates FGF21 Transcription but not of bFGF

Basic fibroblast growth factor and FGF21 promoter sequences analysis showed that two and three putative TCF/LEF binding motifs (AGAAAG) (Schuijers et al., 2014), respectively, were appeared within 3 kb of bFGF and FGF21 promoters. Also, β-catenin interacts with TCF/LEF, and activates the transcription of Wnt signaling genes after β-catenin accumulated in the nucleus (Gordon and Nusse, 2006). Therefore, the possibility of β-catenin complex binding to the bFGF and FGF21 promoters were examined. The two putative binding motifs are located 374 and 518 bp downstream of the start codon of *bFGF*, respectively, while three putative binding motifs are located 976, 1062 and 2269 bp downstream of the start codon of *FGF21*, respectively (Supplementary Figure S8A). To examine whether β-catenin complex directly binds to the putative motifs, ChIP assays were performed using β-catenin antibody. The immunoprecipitated DNA fragments were amplified by five primer pairs which could span P1-P5 regions located within bFGF and FGF21 promoters (Supplementary Figure S8A). The ChIP-PCR data was normalized to input DNA and the results showed that β-catenin complex bound to the P4 and P5 regions in the promoters of FGF21 gene but not of P1-P3 regions in the promoters of bFGF gene (Supplementary Figure S8B).

## Discussion

**FIGURE 8:**
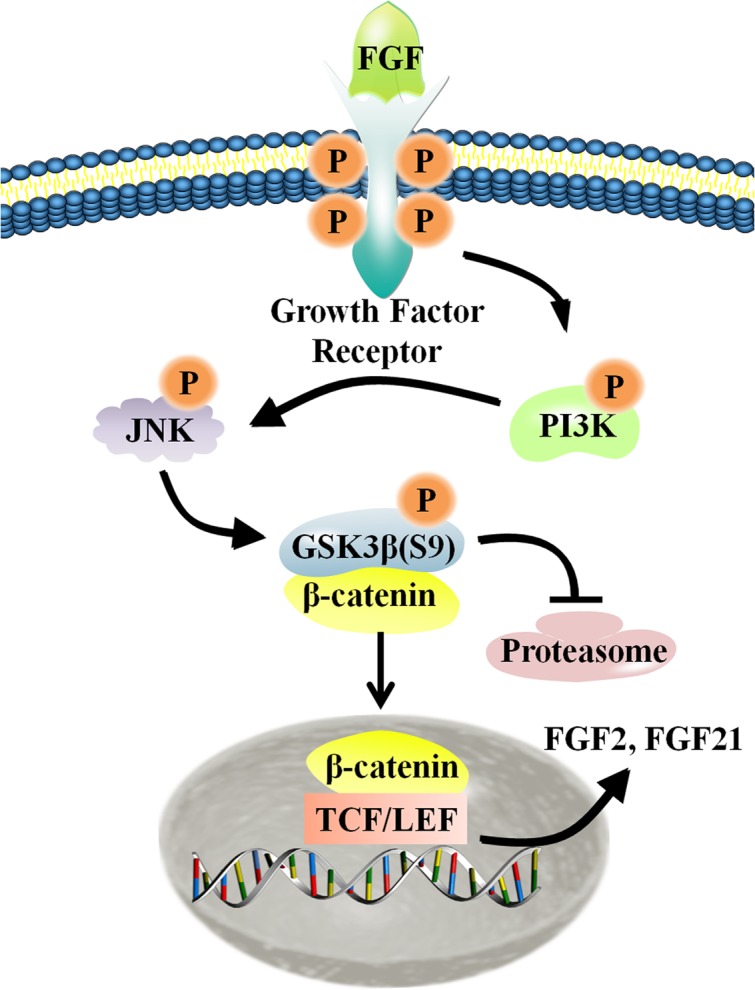
**Schematic representation of the crosstalk between the Wnt and FGF signaling pathways in fibroblasts**. For additional explanation, please see text.

Migration of dermal fibroblasts is crucial for skin wound repair. bFGF is a member of the FGF family of growth factors, and is an efficacious promoter of fibroblast migration. Recent work showed that bFGF activates the PI3K/Rac1/JNK pathway and NFκB-JNK pathway to accelerate cell migration (Xuan et al., 2014, 2016). However, up until now, little information has been available regarding bFGF-meditated control of signaling pathways. Here, we utilized RNA sequencing analysis to demonstrate that Wnt/β-catenin signaling genes are regulated by bFGF. Further analyses indicated that PI3K and JNK function upstream of GSK3β and β-catenin, two principal regulators of Wnt signaling. As the phosphorylation of GSK3β might be indirectly stimulated by MAP kinase, including JNK, we investigated the possibility of a JNK mediated increase in the GSK3β phosphorylation level in bFGF treated fibroblasts (Stambolic and Woodgett, 1994). Coincidentally, exogenous bFGF-mediated stimulation of fibroblasts apparently activated the PI3K/JNK pathway, which in turn phosphorylated GSK3β and positively regulates β-catenin shuttling between the cytoplasm and the nucleus (**Figure 8**). The translocation of β-catenin from the cytoplasm into the nucleus can then activate the downstream transcriptional factors, TCF/LEF, promoting the transcription of Wnt target genes such as *FZD8* and *Wnt3a*, and also inducing the expression of *bFGF* and *FGF21*.

Basic fibroblast growth factor, LiCl, and IM-12 significantly accelerated cell migration. On the other hand, the Wnt inhibitors, IWR-1 and XAV-939, delayed cell migration, and bFGF somewhat attenuated the effects from IWR-1, which is consistent with previous reports (Lam et al., 2011). For LiCl, millimolar concentrations were applied in many research, which play significant roles to study cell proliferation, differentiation, migration (Teng et al., 2010; Azimian-Zavareh et al., 2012). In contrast to the above reports, micromolar concentrations of LiCl were used in some studies, which indicated that low concentrations of LiCl could be effective therapeutic doses in certain conditions (Nakamura et al., 2003; Zarse et al., 2011; Huang et al., 2015; Erguven et al., 2016; Xu et al., 2016). For instance, Erguven et al found that low concentrations of LiCl significantly increased cell number, while high concentrations of LiCl significantly decreased cell number. In our research, LiCl was tested at a concentration ranging from 0.5 to 10 μM in the cell migration. Base on the results, 1 μM LiCl was the more appropriate concentration to enhance migration by inhibited phosphorylation of GSK3β at Ser9, which results in β-catenin stabilization and leads to enhanced Wnt signaling (Fei et al., 2011). Therefore, 1 μM LiCl was applied for further experiments.

At the protein level, bFGF activates the PI3K/JNK pathway, leading to phosphorylation of GSK3β at Ser^9^. Normally, GSK3β maintains low cytoplasmic expression of β-catenin via phosphorylation. Phosphorylated β-catenin can be ubiquitinated through the F-box-containing protein, beta-transducin repeat containing protein, an ubiquitin E3 ligase, and then degraded by the proteasome (Aberle et al., 1997; Kitagawa et al., 1999). Thus, inactivation of GSK3β stabilizes β-catenin and increases its nuclear content. As described above, bFGF-mediated activation of Wnt signaling was apparent herein both *in vitro* and *in vivo*, triggering the migration of fibroblasts and concomitant wound healing. In our previous work, we found that ubiquitination-related genes (OTUB1, Peli1, UBFD1) were altered by bFGF stimulation observed by RNA-Seq assay, indicating that bFGF might not only inactivate GSK3β, but also reduce proteasome-mediated degradation of β-catenin (Xuan et al., 2016).

Also, previous studies found that β-catenin inhibits the proliferation of murine melanoblasts and human melanoma cells (Chien et al., 2009; Luciani et al., 2011), and may play conflicting roles in the metastatic spread of melanoma, repressing migration while promoting metastasis (Gallagher et al., 2013). However, in our study, β*-catenin* suppression significantly delayed the migration of NIH3T3 cells, supporting a pro-migratory role for β-catenin. Furthermore, IWR treatment showed a similar effect to β*-catenin* knock-down. Interestingly, bFGF partially overcame the inhibitory actions of both β*-catenin* repression and IWR-1. Moreover, *a prior* study showed that β-catenin promotes migration, invasion, and proliferation in colorectal, liver, lung, and breast cancers (Gallagher et al., 2013). Combined with our observations, it is concluded that the pro-migratory effect induced by bFGF was mainly mediated by Wnt/β-catenin pathway, notwithstanding the effect contributed by other signaling pathways (Xuan et al., 2014, 2016).

Notably, *FGF21* expression levels were negatively influenced by the down-regulation of β*-catenin*, similar to *bFGF* transcription levels. FGF21 primarily activates FGFR1c, an isoform of FGFR1, and exhibits a preference for the β-klotho co-receptor (Yie et al., 2012; Belov and Mohammadi, 2013). In the present study, FGF21, like bFGF, promoted normal skin fibroblast migration, and increased the migration speed of β*-catenin*-suppressing fibroblasts. β*-klotho* expression was also detected in skin fibroblasts (data not shown), supporting a role for FGF21 in the skin wound healing. In addition, Western blot analysis showed that exogenous FGF21 treatment activated Wnt signaling in fibroblasts to the same extent as exogenous bFGF. These results indicate that acceleration of fibroblast migration through the PI3K/JNK/GSK3β/β-catenin signaling cascade acts in a feedback loop to regulate the expression of both *bFGF* and *FGF21*. However, promoter sequence analysis revealed that the putative β-catenin-TCF4 transcription factor complex binding motifs appeared in the promoter of *FGF21*, but not in *bFGF* (Matsumoto et al., 2014). For further study, it would be extremely interesting to find the reason that β-catenin did not bind the P1-P3 regions in the promoters of bFGF. In addition, TCF4/β-catenin complex directly regulate *FGF21* transcription by ChIP assay, implying that constitutive activation of FGF21 might be important in cell migration process as well as bFGF. Furthermore, FGF21 treatments still accelerated the migration of β*-catenin*-suppressing fibroblast cells comparing with that of the control groups. It may be attributed to the incomplete suppression of endogenous β*-catenin* or activation of other downstream signaling, which is able to partially rescue the cell migration.

Based on the present results and previous works, our analyses provide evidence for the following conclusions: (1) Wnt signaling plays a role in FGF-mediated fibroblast migration; (2) PI3K/JNK acts at the downstream of FGF signaling to positively regulate GSK3β/β-catenin activity; (3) a new role for FGF21 is identified in fibroblast migration; and (4) β-catenin also acts downstream of FGF signaling, but in turn activates transcription of *bFGF* and *FGF21*, two FGF family member genes. Treatment with bFGF and FGF21 partially recovered the delayed cell migration rates in response to reduced β*-catenin* expression, suggesting that β-catenin-facilitated promotion of cell migration stems from transcriptional activation of *bFGF* and *FGF21*, at least in part. Nevertheless, because β-catenin also regulates the transcription of a number of other genes, further analyses are required to identify additional components involved in fibroblast migration during wound healing.

## Author Contributions

XW, YX, YZ, CS, and XL proposed and designed experiments and/or interpreted data; YZ, CS, WHC, YS, and LC performed experiments; HW and NS contributed essential reagents and tools; WTC, TW, CN, and JS participated in writing; WTC and JYS contributed essential reagents and tools and gave conceptual advice; ZZ gave conceptual advice and interpreted data; YX contributed essential reagents and tools, gave conceptual advice and critically revised the paper; LJ supervised the project, interpreted data and wrote the paper.

## Conflict of Interest Statement

The authors declare that the research was conducted in the absence of any commercial or financial relationships that could be construed as a potential conflict of interest.

## Abbreviations

FGF: basic fibroblast growth factor
ChIP: chromatin immunoprecipitation
DMSO: dimethyl sulfoxide
ECM: extracellular matrix
ELISA: enzyme-linked immunosorbent assay
FBS: fetal bovine serum
FGFR: fibroblast growth factor receptor
FZD8: Frizzled 8
GAPDH: glyceraldehyde-3-phosphate dehydrogenase
GO: gene ontology
GSK-3β: glycogen synthase kinase 3β
JNK: c-Jun N-terminal kinase
LiCl: lithium chloride
PBS: phosphate buffered saline
PI3K: phosphatidylinositol 3-kinase
qRT-PCR: quantitative real-time polymerase chain reaction
RNA-Seq: RNA-sequencing
siRNA: small interfering RNA
TCF7: T-cell factor 7

## References

[B1] Aberle H. Bauer A. Stappert J. Kispert A. Kemler R. (1997). beta-catenin is a target for the ubiquitin-proteasome pathway. *EMBO J.* 16 3797–3804. 10.1093/emboj/16.13.3797 9233789 PMC1170003

[B2] Aman A. Piotrowski T. (2008). Wnt/β-catenin and fgf signaling control collective cell migration by restricting chemokine receptor expression. *Dev. Cell* 11 749–761. 10.1016/j.devcel.2008.10.002 19000839

[B3] Amini-Nik S. Cambridge E. Yu W. Guo A. Whetstone H. Nadesan P. (2014). β-catenin–regulated myeloid cell adhesion and migration determine wound healing. *J. Clin. Invest.* 124 2599–2610. 10.1172/JCI62059 24837430 PMC4089463

[B4] Azimian-Zavareh V. Hossein G. Janzamin E. (2012). Effect of lithium chloride and antineoplastic drugs on survival and cell cycle of androgen-dependent prostate cancer LNCap cells. *Indian J. Pharmacol.* 44 714–721. 10.4103/0253-7613.103265 23248400 PMC3523498

[B5] Badman M. K. Pissios P. Kennedy A. R. Koukos G. Flier J. S. Maratos-Flier E. (2007). Hepatic fibroblast growth factor 21 is regulated by PPARalpha and is a key mediator of hepatic lipid metabolism in ketotic states. *Cell Metab.* 5 426–437. 10.1016/j.cmet.2007.05.002 17550778

[B6] Belov A. A. Mohammadi M. (2013). Molecular mechanisms of fibroblast growth factor signaling in physiology and pathology. *Cold Spring Harb. Perspect. Biol.* 5 a015958. 10.1101/cshperspect.a015958 PMC3660835 23732477

[B7] Bergmann C. Akhmetshina A. Dees C. Palumbo K. Zerr P. Beyer C. (2011). Inhibition of glycogen synthase kinase 3β induces dermal fibrosis by activation of the canonical Wnt pathway. *Ann. Rheum. Dis.* 70 2191–2198. 10.1136/ard.2010.147140 21873331

[B8] Bianchi M. De Lucchini S. Marin O. Turner D. Hanks S. Villa-Moruzzi E. (2005). Regulation of FAK Ser-722 phosphorylation and kinase activity by GSK3 and PP1 during cell spreading and migration. *Biochem. J.* 391 359–370. 10.1042/BJ20050282 15975092 PMC1276935

[B9] Boku S. Nakagawa S. Toda H. Kato A. Takamura N. Omiya Y. (2013). ROCK2 regulates bFGF-induced proliferation of SH-SY5Y cells through GSK-3β and β-catenin pathway. *Brain Res.* 1492 7–17. 10.1016/j.brainres.2012.11.034 23211630

[B10] Brack A. S. Conboy M. J. Roy S. Lee M. Kuo C. J. Keller C. (2007). Increased Wnt signaling during aging alters muscle stem cell fate and increases fibrosis. *Science* 317 807–810. 10.1126/science.1144090 17690295

[B11] Cai T. Sun D. Q. Duan Y. Wen P. Dai C. S. Yang J. W. (2016). WNT/β-catenin signaling promotes VSMCs to osteogenic transdifferentation and calcification through directly modulating Runx2 gene expression. *Exp. Cell Res.* 345 206–217. 10.1016/j.yexcr.2016.06.007 27321958

[B12] Chien A. J. Moore E. C. Lonsdorf A. S. Kulikauskas R. M. Rothberg B. G. Berger A. J. (2009). Activated Wnt/beta-catenin signaling in melanoma is associated with decreased proliferation in patient tumors and a murine melanoma model. *Proc. Natl. Acad. Sci. U.S.A.* 27 1193–1198. 10.1073/pnas.0811902106 PMC2626610 19144919

[B13] Clevers H. (2006). Wnt/β-catenin signaling in development and disease. *Cell* 127 469–480. 10.1016/j.cell.2006.10.018 17081971

[B14] Clevers H. Nusse R. (2012). Wnt/beta-catenin signaling and disease. *Cell* 8 1192–1205. 10.1016/j.cell.2012.05.012 22682243

[B15] Cohen P. Frame S. (2001). The renaissance of GSK3. *Nat. Rev. Mol. Cell Biol.* 2 769–776. 10.1038/35096075 11584304

[B16] Consortium G. O. (2006). The gene ontology (GO) project in 2006. *Nucleic Acids Res.* 34 D322–D326. 10.1093/nar/gkj021 16381878 PMC1347384

[B17] Costa C. Ebi H. Martini M. Beausoleil S. A. Faber A. C. Jakubik C. T. (2014). Measurement of PIP3 levels reveals an unexpected role for p110β in early adaptive responses to p110α-specific inhibitors in luminal breast cancer. *Cancer Cell* 27 97–108. 10.1016/j.ccell.2014.11.007 25544637 PMC4745884

[B18] Draghici S. Khatri P. Tarca A. L. Amin K. Done A. Voichita C. (2007). A systems biology approach for pathway level analysis. *Genome Res.* 17 1537–1545. 10.1101/gr.6202607 17785539 PMC1987343

[B19] Dunnick J. Brix A. Cunny H. Vallant M. Shockley K. (2012). Characterization of polybrominated diphenyl ether toxicity in wistar han rats and use of liver microarray data for predicting disease susceptibilities. *Toxicol. Pathol.* 40 93–106. 10.1177/0192623311429973 22267650 PMC4816085

[B20] Dupuy D. Bertin N. Hidalgo C. A. Venkatesan K. Tu D. Lee D. (2007). Genome-scale analysis of in vivo spatiotemporal promoter activity in *Caenorhabditis elegans*. *Nat. Biotechnol.* 25 663–668. 10.1038/nbt1305 17486083

[B21] Dvorak P. Hampl A. (2005). Basic fibroblast growth factor and its receptors in human embryonic stem cells. *Folia Histochem. Cytobiol.* 43 203–208. 16382885

[B22] Engelman J. A. Luo J. Cantley L. C. (2006). The evolution of phosphatidylinositol 3-kinases as regulators of growth and metabolism. *Nat. Rev. Genet.* 7 606–619. 10.1038/nrg1879 16847462

[B23] Enoch S. Price P. (2004). Cellular, molecular and biochemical differences in the pathophysiology of healing between acute wounds, chronic wounds and wounds in the aged. *World Wide Wounds.* Available at: http://www.worldwidewounds.com/2004/august/Enoch/Pathophysiology-Of-Healing.html

[B24] Epstein F. H. Singer A. J. Clark R. A. (1999). Cutaneous wound healing. *New England J. Med.* 341 738–746. 10.1056/NEJM199909023411006 10471461

[B25] Erguven M. Oktem G. Kara A. N. Bilir A. (2016). Lithium chloride has a biphasic effect on prostate cancer stem cells and a proportional effect on midkine levels. *Oncol. Lett.* 12 2948–2955. 27703531 10.3892/ol.2016.4946 PMC5038888

[B26] Etienne-Manneville S. Hall A. (2003). Cdc42 regulates GSK-3β and adenomatous polyposis coli to control cell polarity. *Nature* 421 753–756. 10.1038/nature01423 12610628

[B27] Fei Y. Xiao L. Doetschman T. Coffin D. J. Hurley M. M. (2011). Fibroblast growth factor 2 stimulation of osteoblast differentiation and bone formation is mediated by modulation of the Wnt signaling pathway. *J. Biol. Chem.* 25 40575–40583. 10.1074/jbc.M111.274910 PMC3220493 21987573

[B28] Fukui M. Choi H. J. Zhu B. T. (2010). Mechanism for the protective effect of resveratrol against oxidative stress-induced neuronal death. *Free Radic. Biol. Med.* 1 800–813. 10.1016/j.freeradbiomed.2010.06.002 PMC2938064 20542495

[B29] Gallagher S. J. Rambow F. Kumasaka M. Champeval D. Bellacosa A. Delmas V. (2013). Beta-catenin inhibits melanocyte migration but induces melanoma metastasis. *Oncogene* 32 2230–2238. 10.1038/onc.2012.229 22665063 PMC3637425

[B30] Gordon M. D. Nusse R. (2006). Wnt signaling: multiple pathways, multiple receptors, and multiple transcription factors. *J. Biol. Chem.* 11 22429–22433. 10.1074/jbc.R600015200 16793760

[B31] Harris E. S. Nelson W. J. (2010). Adenomatous polyposis coli regulates endothelial cell migration independent of roles in β-Catenin signaling and cell–cell adhesion. *Mol. Biol. Cell* 21 2611–2623. 10.1091/mbc.E10-03-0235 20519433 PMC2912348

[B32] Hu Y. Poopalasundaram S. Graham A. Bouloux P. M. (2013). GnRH neuronal migration and olfactory bulb neurite outgrowth are dependent on FGF receptor 1 signaling, specifically via the PI3K p110αisoform in chick embryo. *Endocrinology* 154 388–399. 10.1210/en.2012-1555 23150492

[B33] Huang C. K. Yu T. de la Monte S. M. Wands J. R. Derdak Z. Kim M. (2015). Restoration of Wnt/beta-catenin signaling attenuates alcoholic liver disease progression in a rat model. *J. Hepatol.* 63 191–198. 10.1016/j.jhep.2015.02.030 25724365 PMC4475483

[B34] Jeong M. H. Ho S. M. Vuong T. A. Jo S. B. Liu G. Z. Aaronson S. A. (2014). Cdo supresses canonical Wnt signaling via interaction with Lrp6 thereby promoting neuronal differentiation. *Nat. Commun.* 9 5455–5468. 10.1038/ncomms6455 PMC4412020 25406935

[B35] Kanazawa S. Fujiwara T. Matsuzaki S. Shingaki K. Taniguchi M. Miyata S. (2010). bFGF regulates PI3-kinase-Rac1-JNK pathway and promotes fibroblast migration in wound healing. *PLoS ONE* 5:e12228 10.1371/journal.pone.0012228 PMC2923192 20808927

[B36] Kitagawa M. Hatakeyama S. Shirane M. Matsumoto M. Ishida N. Hattori K. (1999). An F-box protein, FWD1, mediates ubiquitin-dependent proteolysis of beta-catenin. *EMBO J.* 4 2401–2410. 10.1093/emboj/18.9.2401 PMC1171323 10228155

[B37] Klein P. S. Melton D. A. (1996). A molecular mechanism for the effect of lithium on development. *Proc. Natl. Acad. Sci. U.S.A.* 93 8455–8459. 10.1073/pnas.93.16.8455 8710892 PMC38692

[B38] Lam A. P. Flozak A. S. Russell S. Wei J. Jain M. Mutlu G. M. (2011). Nuclear β-catenin is increased in systemic sclerosis pulmonary fibrosis and promotes lung fibroblast migration and proliferation. *Am. J. Respir. Cell Mol. Biol.* 45 915–922. 10.1165/rcmb.2010-0113OC 21454805 PMC3262680

[B39] Liang Q. Zhong L. Zhang J. Wang Y. Bornstein S. R. Triggle C. R. (2014). FGF21 maintains glucose homeostasis by mediating the cross talk between liver and brain during prolonged fasting. *Diabetes Metab. Res. Rev.* 63 4064–4075. 10.2337/db14-0541 25024372

[B40] Lin Z. Tian H. Lam K. S. Lin S. Hoo R. C. Konishi M. (2013). Adiponectin mediates the metabolic effects of FGF21 on glucose homeostasis and insulin sensitivity in mice. *Cell Metab.* 17 779–789. 10.1016/j.cmet.2013.04.005 23663741

[B41] Lin Z. Wu F. Lin S. Pan X. Jin L. Lu T. (2014). Adiponectin protects against acetaminophen-induced mitochondrial dysfunction and acute liver injury by promoting autophagy in mice. *J. Hepatol.* 61 825–831. 10.1016/j.jhep.2014.05.033 24882054

[B42] Luciani F. Champeval D. Herbette A. Denat L. Aylaj B. Martinozzi S. (2011). Biological and mathematical modeling of melanocyte development. *Development* 138 3943–3954. 10.1242/dev.067447 21862558

[B43] Martin P. (1997). Wound healing–aiming for perfect skin regeneration. *Science* 276 75–81. 10.1126/science.276.5309.75 9082989

[B44] Matsumoto S. Fujii S. Sato A. Ibuka S. Kagawa Y. Ishii M. (2014). A combination of Wnt and growth factor signaling induces Arl4c expression to form epithelial tubular structures. *EMBO J.* 1 702–718. 10.1002/embj.201386942 PMC4000088 24562386

[B45] Moon R. T. Kohn A. D. De Ferrari G. V. Kaykas A. (2004). WNT and β-catenin signalling: diseases and therapies. *Nat. Rev. Genet.* 5 691–701. 10.1038/nrg1427 15372092

[B46] Nakamura T. Sano M. Song Y. Z. Schneider M. D. (2003). A Wnt-and beta-catenin-dependent pathway for mammalian cardiac myogenesis. *Proc. Natl. Acad. Sci. U.S.A.* 100 5834–5839. 10.1073/pnas.0935626100 12719544 PMC156287

[B47] Nishimura T. Nakatake Y. Konishi M. Itoh N. (2000). Identification of a novel FGF, FGF-21, preferentially expressed in the liver. *Biochim. Biophys. Acta.* 1492 203–206. 10.1016/S0167-4781(00)00067-1 10858549

[B48] Pazyar N. Yaghoobi R. Rafiee E. Mehrabian A. Feily A. (2014). Skin wound healing and phytomedicine: a review. *Skin Pharmacol. Physiol.* 27 303–310. 10.1159/000357477 24993834

[B49] Salic A. Lee E. Mayer L. Kirschner M. W. (2000). Control of β-catenin stability: reconstitution of the cytoplasmic steps of the wnt pathway in Xenopus egg extracts. *Mol. Cell* 5 523–532. 10.1016/S1097-2765(00)80446-3 10882137

[B50] Schuijers J. Mokry M. Hatzis P. Cuppen E. Clevers H. (2014). Wnt-induced transcriptional activation is exclusively mediated by TCF/LEF. *EMBO J.* 33 146–156. 10.1002/embj.201385358 24413017 PMC3989608

[B51] Shimizu H. Julius M. A. Giarre M. Zheng Z. L. Brown A. M. (1997). Transformation by Wnt family proteins correlates with regulation of β-catenin. *Cell Growth Differ.* 8 1349–1358. 9419423

[B52] Shtutman M. Zhurinsky J. Simcha I. Albanese C. D’Amico M. Pestell R. (1999). The cyclin D1 gene is a target of the beta-catenin/LEF-1 pathway. *Proc. Natl. Acad. Sci. U.S.A.* 11 5522–5527. 10.1073/pnas.96.10.5522 PMC21892 10318916

[B53] Singh R. Bhasin S. Braga M. Artaza J. N. Pervin S. Taylor W. E. (2009). Regulation of myogenic differentiation by androgens: cross talk between androgen receptor/β-catenin and follistatin/transforming growth factor-β signaling pathways. *Endocrinology* 150 1259–1268. 10.1210/en.2008-0858 18948405 PMC2654730

[B54] Stambolic V. Woodgett J. R. (1994). Mitogen inactivation of glycogen synthase kinase-3 beta in intact cells via serine 9 phosphorylation. *Biochem. J.* 303(Pt. 3), 701–704. 10.1042/bj3030701 7980435 PMC1137602

[B55] Sun T. Rodriguez M. Kim L. (2009). Glycogen synthase kinase 3 in the world of cell migration. *Dev. Growth Differ.* 51 735–742. 10.1111/j.1440-169X.2009.01141.x 19891643

[B56] Teng Y. Wang X. Wang Y. Ma D. (2010). Wnt/beta-catenin signaling regulates cancer stem cells in lung cancer A549 cells. *Biochem. Biophys. Res. Commun.* 12 373–379. 10.1016/j.bbrc.2010.01.028 20074550

[B57] Thamm O. C. Theodorou P. Stuermer E. Zinser M. J. Neugebauer E. A. Fuchs P. C. (2013). Adipose-derived stem cells and keratinocytes in a chronic wound cell culture model: the role of hydroxyectoine. *Int. Wound J.* 12 387–396. 10.1111/iwj.12120 23841674 PMC7950684

[B58] Wagner W. Wehrmann M. (2007). Differential cytokine activity and morphology during wound healing in the neonatal and adult rat skin. *J. Cell Mol. Med.* 11 1342–1351. 10.1111/j.1582-4934.2007.00037.x 18205704 PMC4401296

[B59] Xu Y. Z. Wang Q. Li D. S. Wu Z. H. Li D. W. Lu K. L. (2016). Protective effect of Lithium chloride against hypoglycemia-induced apoptosis in beuronal PC21 cell. *Neuroscience* 330 100–108. 10.1016/j.neuroscience.2016.05.047 27241942

[B60] Xuan Y. Chi L. Tian H. Cai W. Sun C. Wang T. (2016). The activation of the NF-kappaB-JNK pathway is independent of the PI3K-Rac1-JNK pathway involved in the bFGF-regulated human fibroblast cell migration. *J. Dermatol. Sci.* 82 28–37. 10.1016/j.jdermsci.2016.01.003 26829882

[B61] Xuan Y. H. Huang B. B. Tian H. S. Chi L. S. Duan Y. M. Wang X. (2014). High-glucose inhibits human fibroblast cell migration in wound healing via repression of bFGF-regulating JNK phosphorylation. *PLoS ONE* 9:e108182 10.1371/journal.pone.0108182 PMC4171528 25244316

[B62] Yie J. Wang W. Deng L. Tam L. T. Stevens J. Chen M. M. (2012). Understanding the physical interactions in the FGF21/FGFR/beta-Klotho complex: structural requirements and implications in FGF21 signaling. *Chem. Biol. Drug Design.* 79 398–410. 10.1111/j.1747-0285.2012.01325.x 22248288

[B63] Zarse K. Terao T. Tian J. Iwata N. Ishii N. Ristow M. (2011). Low-dose lithium uptake promotes longevity in humans and metazoans. *Eur. J. Nutr.* 50 387–389. 10.1007/s00394-011-0171-x 21301855 PMC3151375

[B64] Zhang H. Zhao D. Wang Z. Zheng D. (2010). Diazoxide preconditioning alleviates caspase-dependent and caspase-independent apoptosis induced by anoxia-reoxygenation of PC12 cells. *J. Biochem.* 148 413–421. 10.1093/jb/mvq074 20616381

[B65] Zittermann S. I. Issekutz A. C. (2006). Basic fibroblast growth factor (bFGF, FGF-2) potentiates leukocyte recruitment to inflammation by enhancing endothelial adhesion molecule expression. *Am. J. Pathol.* 168 835–846. 10.2353/ajpath.2006.050479 16507899 PMC1606526

