# Supplementary material for: Feedback Activation of Basic Fibroblast Growth Factor Signaling via the Wnt/β-Catenin Pathway in Skin Fibroblasts
Source: Front Pharmacol. 2017 Feb 3;8:32. doi: 10.3389/fphar.2017.00032 (PMC5289949; doi:10.3389/fphar.2017.00032)
Supplement: Supplementary file 1 [file Data_Sheet_1.DOCX]

Supplementary Material

**Feedback activation of basic fibroblast growth factor signaling via the Wnt/β-catenin pathway in skin fibroblasts**

**Xu Wang^1#^, Yuting Zhu^2#^, Congcong Sun^1#^, Tao Wang^1^, Yingjie Shen^1^, Wanhui Cai^1^, Jia Sun^1^, Lisha Chi^1^, Haijun Wang^3^, Na Song^3^, Chao Niu^1^, Jiayi Shen^1^, Weitao Cong^1^, Zhongxin Zhu^1^, Yuanhu Xuan^1^*, Xiaokun Li^1^*, Litai Jin^1^***

## * Correspondence: Litai Jin: jin_litai@126.com; Tel: +86-577-86699790;

## Xiaokun Li: proflxk@163.com; Tel: +86-577-86699790;

## Yuanhu Xuan: [yhxuan@wzmc.edu.cn](mailto:yhxuan@wzmc.edu.cn); Tel: +86-577-86699790

**Supplementary Figures and Tables**

**
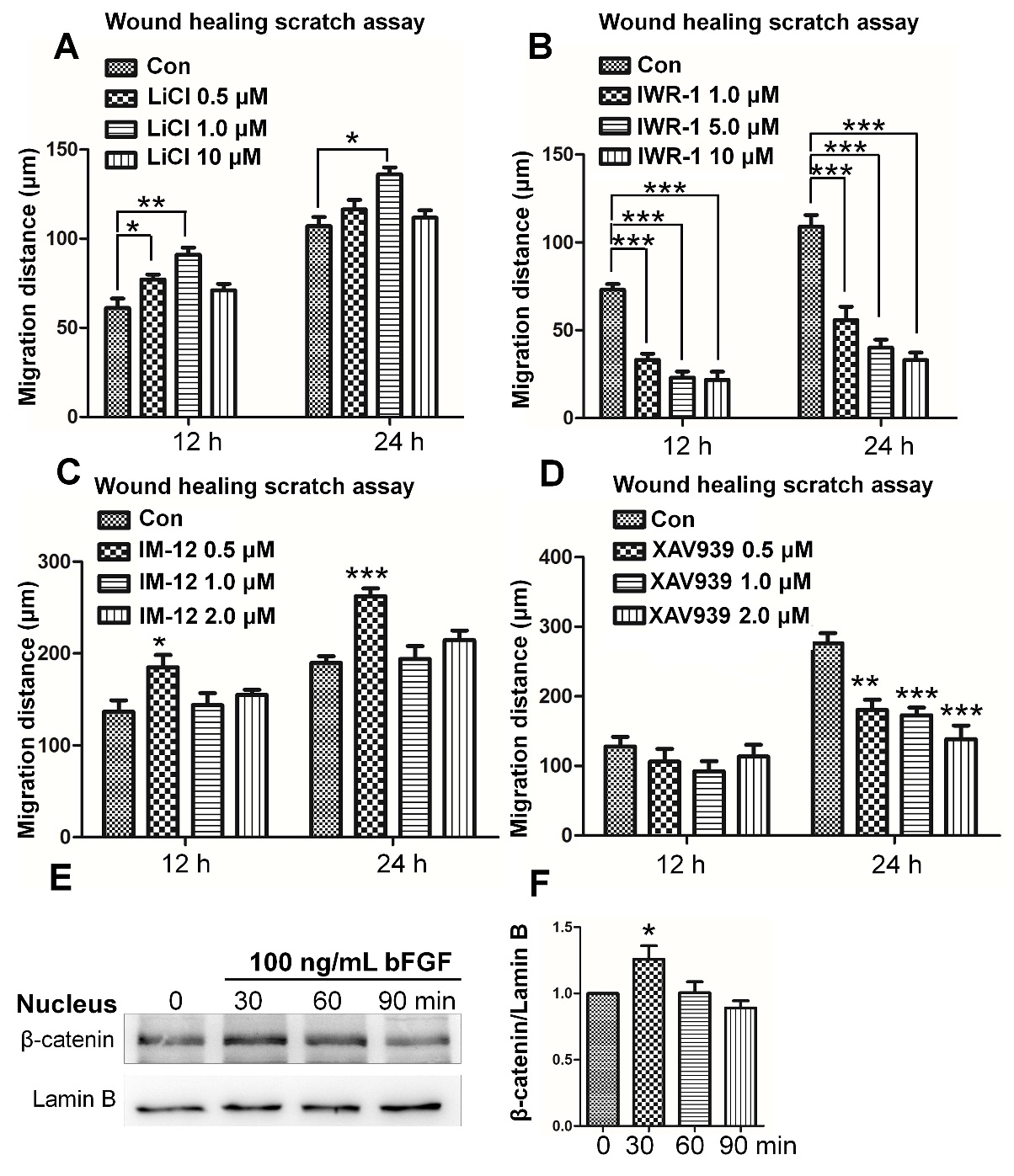
**

**Figure. S1.** The different concentrations of reagents used in testing the cell migration and time-dependent β-catenin expressions after the treatment of bFGF. The cell migration under the different concentrations of LiCl (A), IWR-1 (B), IM-12 (C) and XAV939 (D) were measured by wound healing scratch assay after 24 h. (E) The cells were treated with 100ng/mL bFGF for 30, 60 and 90 min. The levels of β-catenin were analyzed by Western blot analysis. Lamin B was used as the loading control. (F) Densitometry data for β-catenin from the blots shown in (E) were normalized to those of Lamin B. Data represent mean values ± SE of 5 replicates, as compared to the control group (**P<0.05, **P<0.01, ***P<0.001*).


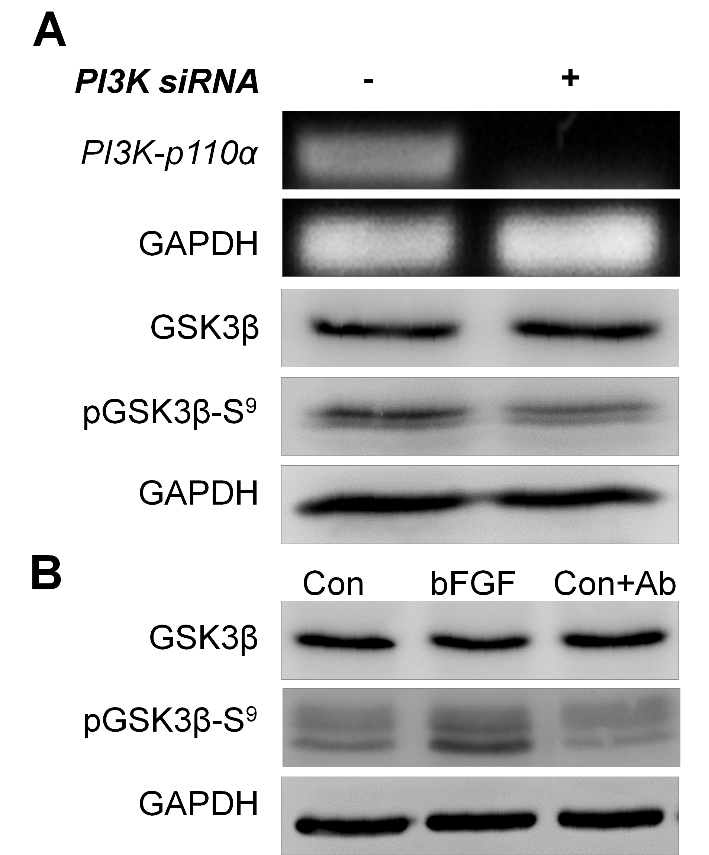


**Figure. S2.** Effects of siRNA-mediated inhibition of PI3K and antibody-mediated neutralization of bFGF on GSK3β phosphorylation. (A) Cells were transfected with or without 40 nM siRNA for 24 h and processed for semi-quantitative RT-PCR of PI3K with the p110α primer. Total cell protein extracts were then prepared and subjected to Western blotting. Phosphorylation levels of GSK3β at Ser^9^ were reduced by siRNA treatment. (B) Western blotting was used to analyze the protein levels of pGSK3β treated with bFGF (100 ng/mL) or anti-bFGF antibody (5 μg/mL) for 30 min.


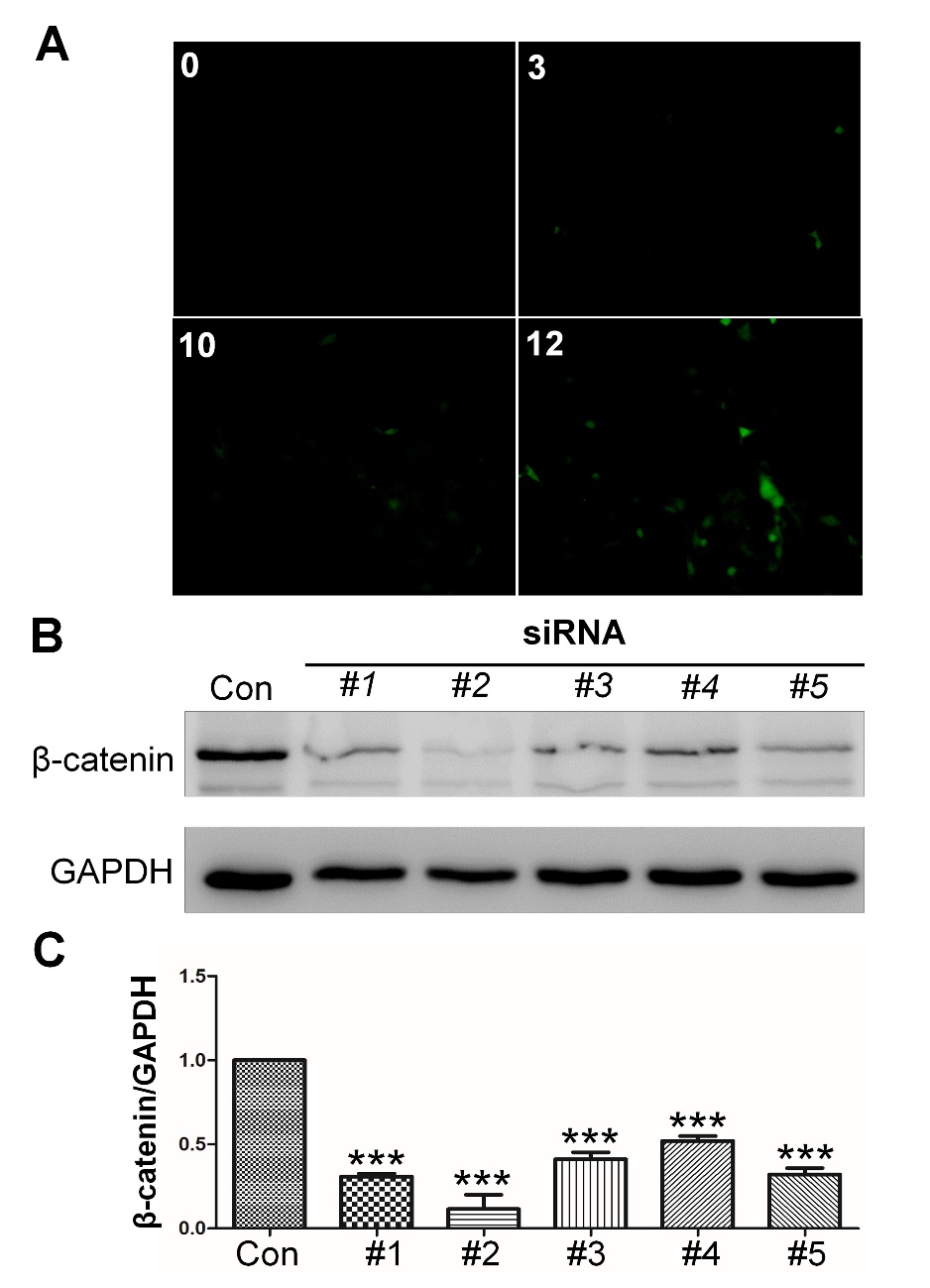


**Figure. S3.** siRNA-mediated inhibition of *β-catenin* in NIH 3T3 cells. (A) NIH3T3 cells were analyzed for GFP expression along with parental (non-infected) NIH 3T3 cells. The lentivirus was used at a volume of 3, 10, or 12 μL, and a titer of 6.51 × 10^5^ IU/μL. (B) Clone groups (*#1–#5*) were harvested from cells transfected with 12 μLof lentivirus for 24 h, and subsequently maintained in regular growth medium for 3 days. Cells were then subjected to Western blot analysis of β-catenin levels. Densitometry data for β-catenin (C) shown in the blot in (B) were normalized to those of GAPDH. Data represent means ± the SE (n = 3 independent experiments; ****P* < 0.001 versus control group; Student’s *t* test).


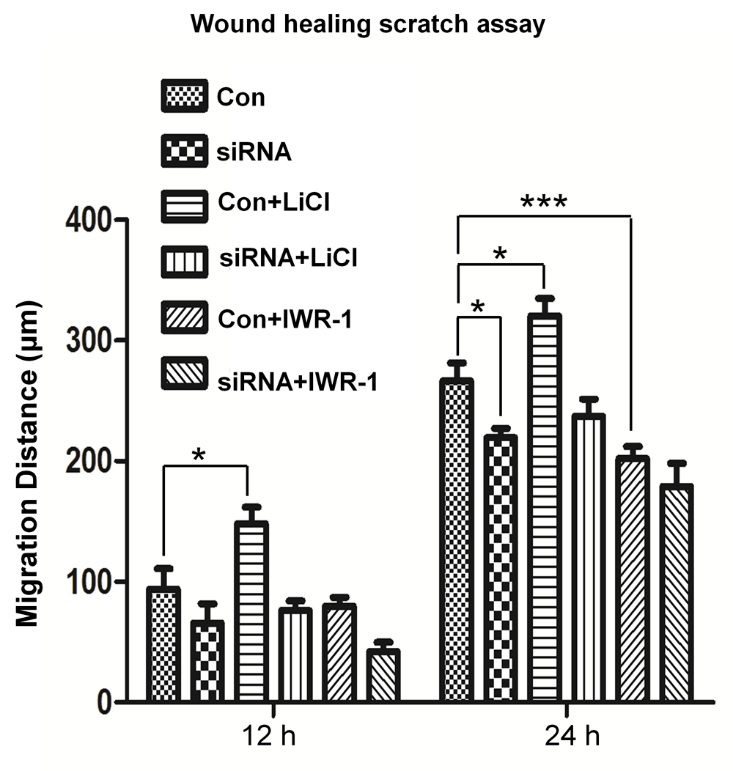


**Figure. S4.** Effects of siRNA-mediated inhibition of *β-catenin* on NIH3T3 cell migration. A wound healing assay was performed in cells transfected with scrambled control siRNA or β-catenin siRNA with or without LiCl (1.0 μM) and IWR-1 (1.0μM). Cell migration distances were measured and plotted (**P*< 0.05, ***P*< 0.01, ****P <0.001*; Student’s *t* test). Con, Control.


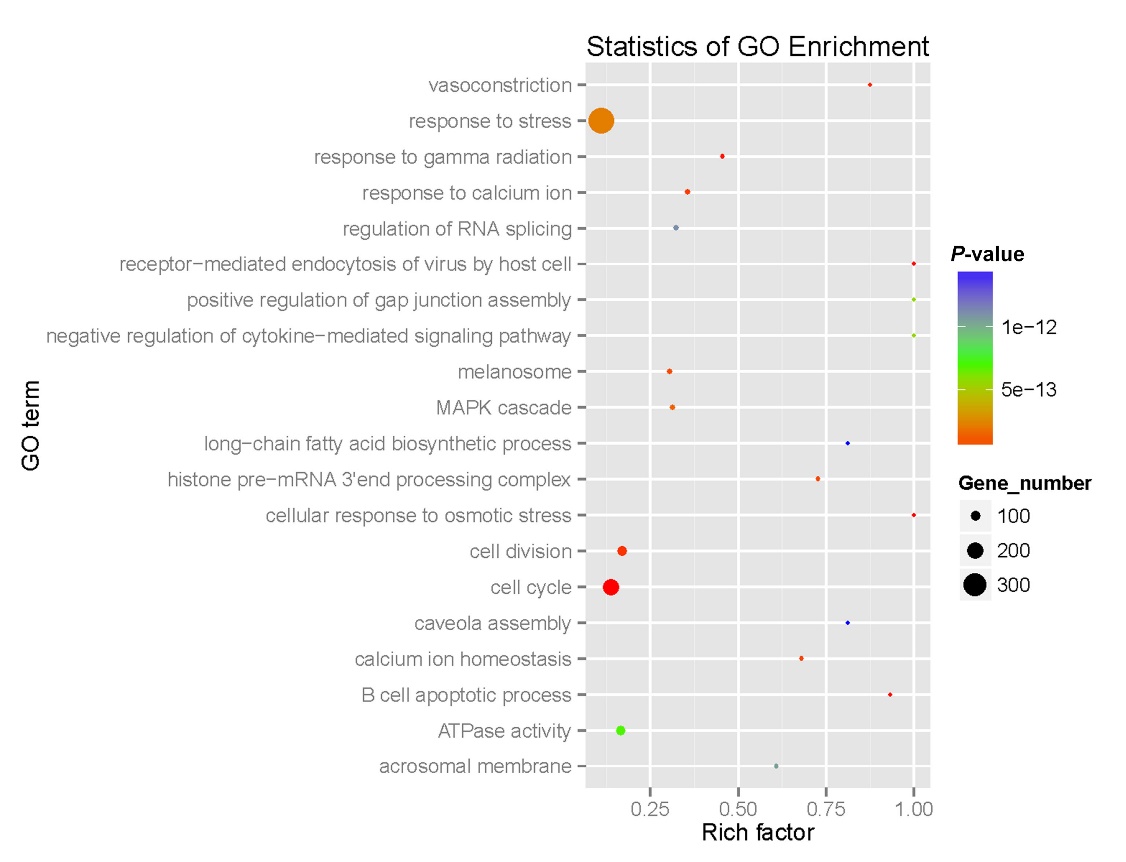


**Figure. S5.** Enrichment of specific GO terms under β-catenin suppression. The circle size represents the number of genes supporting the GO term.

**
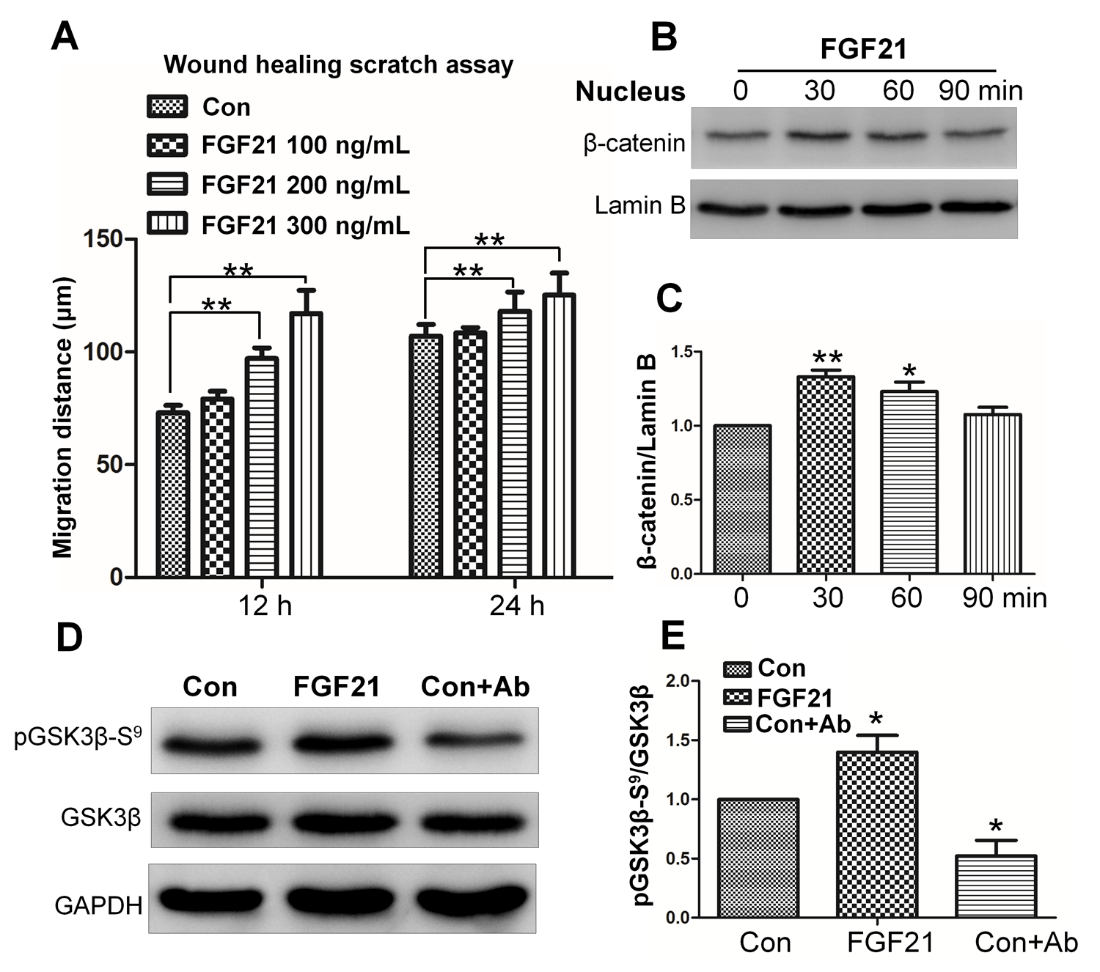
**

**Figure. S6.** The different concentrations of FGF21 used in testing the cell migration and time-dependent β-catenin expressions after the treatment of FGF21.The cell migration under the different concentrations of FGF21 (A) were measured by wound healing scratch assay after 24 h. (B) The cells were treated with 200ng/mL FGF21 for 30, 60 or 90 min. The protein levels of β-catenin were analyzed by Western blot analysis. Lamin B was used as the loading control. (C) Densitometry data for β-catenin from the blots shown in (B) were normalized to those of Lamin B. (D) Western blotting was used to analyze the protein levels of pGSK3β treated with FGF21 (200 ng/mL) or anti-FGF21 antibody (2.5 μg/mL) for 30 min. (E) Densitometry data for pGSK3β from the blots shown in (D) were normalized to those of GAPDH. Data represent mean values ± SE of 5 replicates, as compared to the control group (**P<0.05, **P<0.01,*).

**
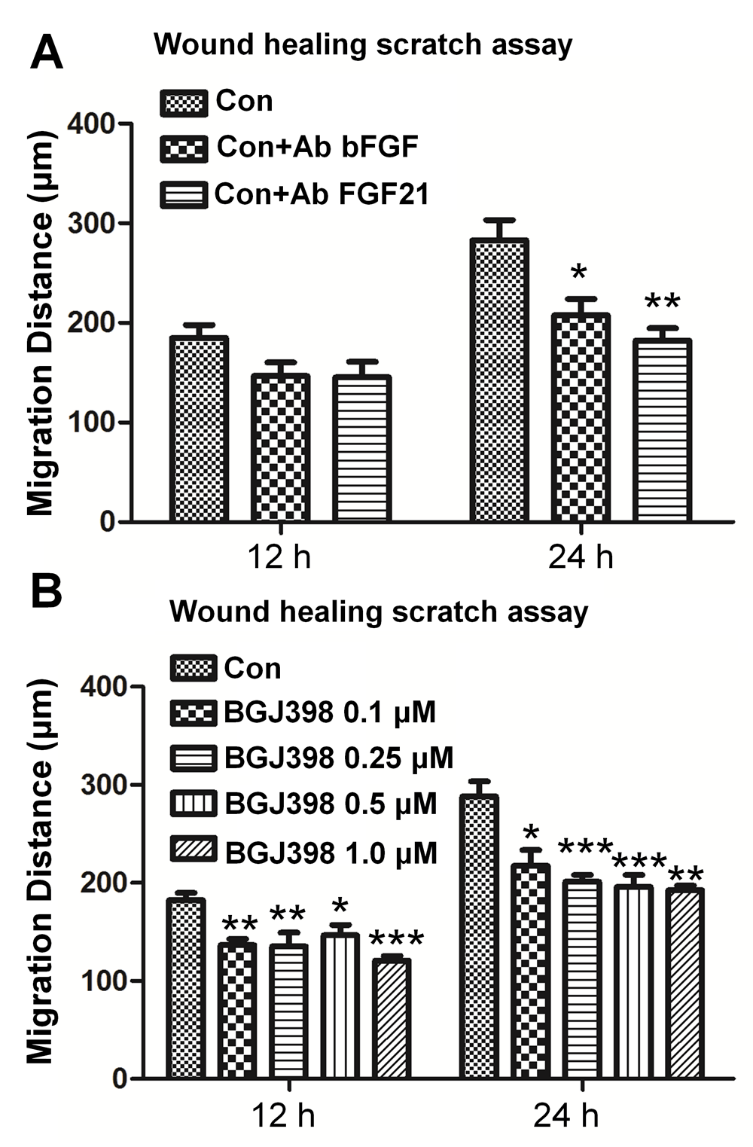
**

**Figure. S7.** Effects of silencing of FGF and the different concentrations of BGJ398, an inhibitor of FGFR used in testing the cell migration in fibroblasts. The cell migration under anti-bFGF antibody (5.0μg/mL), anti-FGF21 antibody (2.5μg/mL) (A) and the different concentrations of BGJ398(B) were measured by wound healing scratch assay after 24 h. Data represent mean values ± SE of 5 replicates, as compared to the control group (**P<0.05, **P<0.01,*****P <0.001*).

**
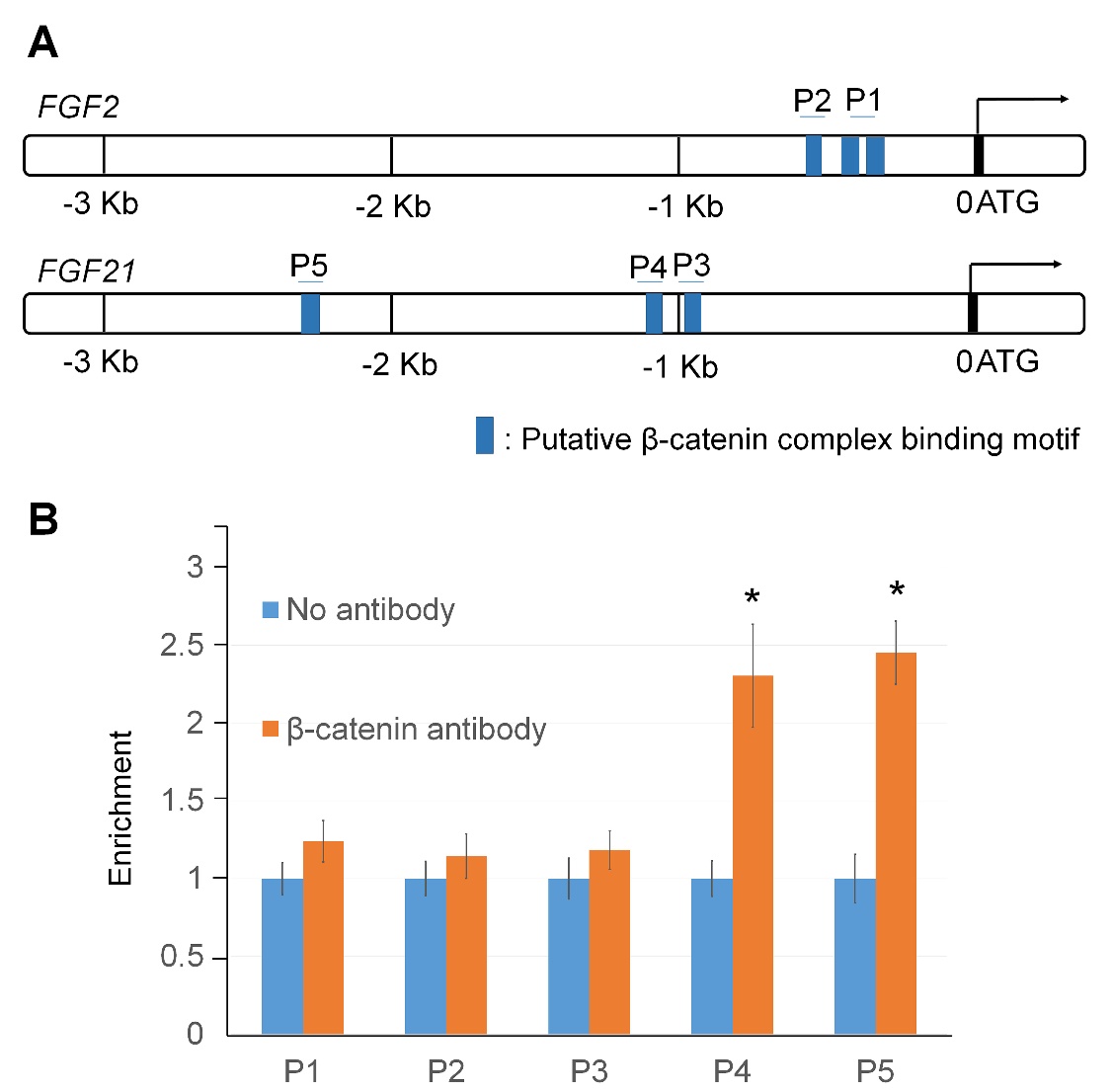
**

**Figure. S8.** β-catenin directly activates *FGF21* transcription but not of *FGF2*. (A) The putative β-catenin complex binding motifs of *FGF2* and *FGF21*. (B) CHIP-PCR performed in NIH3T3 cells, values show fold enrichment over input DNA. Means of 3 individual experiments are shown.

**Table S1.** Primer sequences for qRT-PCR

| Primer | Sequences |
| --- | --- |
| FGF2 F | CAAGAACGGCGGCTTCTTC |
| FGF2 R | GGAAGAAACAGTATGGCCT |
| FGF21 F | GATGACGACCAAGACACTG |
| FGF21 R | CGGCCCTGTAAAGGCTCT |
| Wnt3 F | ATCATAAGGGGCCGCCTGGCGAAGGCTGG |
| Wnt3 R | CTTGCAGGTGTGCACGTCGTAGA |
| Wnt11 F | GAACTGCTCCTCCATTGAGCTC |
| Wnt11 R | GGTATCGGGTCTTGAGGTCAG |
| Wnt3a F | CTCCTCTCGGATACCTCTTAGTG |
| Wnt3a R | ATCCCTCTGCACAGGAGCGT |
| Tcf7 F | CTGCAGACCCCTGACCTCTCT |
| Tcf7 R | ATCCTTGATGCTAGGTTCTGGTGT |
| WNT2B F | AAGGTACATTGGGGCACTGG |
| WNT2B R | CGCGAGTAATAGCGTGGACT |
| FZD8 F | CTGGTGGAGATCCAGTGCTC |
| FZD8 R | TTGTAGTCCATGCACAGCGT |
| m-PIK3CA F | GGCTCTGGAATGCCAGAACTA |
| m-PIK3CA R | CCACCATGATGTGCATCATTCAT |
| GAPDH human F | GACCTGCCGTCTAGAAAAAC |
| GAPDH human R | CTGTAGCCAAATTCGTTGTC |
| GAPDH mouse F | GCCAAGGTCATCCATGACAACT |
| GAPDH mouse R | GAGGGGCCATCCACAGTCTT |
| P1 F | CATCTTCCCACGCTGTCTCG |
| P1 R | CTTTCTCCGCTCCTGCCTTT |
| P2 F | CAAAGCCTGACTTGATCCCTC |
| P2 R | CAGTGCAGCCGGACTCTTC |
| P3 F | CTAAGCAGGGGTTGGTGAGG |
| P3 R | GCGTGTCTGAGGCTTTCTTTC |
| P4 F | ACACCAGCTCAGTTGCTTACAC |
| P4 R | ACTGAAGTCTACACTCCTGGGTCT |
| P5 F | ACACCAGCTCAGTTGCTTACAC |
| P5 R | ACTGAAGTCTACACTCCTGGGTC |

**Table S2.** List of up or down-regulated genes in β-catenin suppressing cells.Fold change (FC), the relative expression level of genes in*β-catenin* knock-down group compared with the control.
